# Association of ADAM family members with proliferation signaling and disease progression in multiple myeloma

**DOI:** 10.1038/s41408-024-01133-4

**Published:** 2024-09-11

**Authors:** Marietheres Evers, Thorsten Stühmer, Martin Schreder, Torsten Steinbrunn, Martina Rudelius, Franziska Jundt, Regina Ebert, Tanja Nicole Hartmann, Ralf Christian Bargou, Andreas Rosenwald, Ellen Leich

**Affiliations:** 1 Institute of Pathology, University of Würzburg, Würzburg, Germany; 2 Comprehensive Cancer Center Mainfranken, University Hospital of Würzburg, Würzburg, Germany; 3 First Department of Medicine, Klinik Ottakring, Vienna, Austria; 4 Department of Medical Oncology, Dana-Farber Cancer Institute, Harvard Medical School, Boston, MA USA; 5 Department of Internal Medicine II, University Hospital of Würzburg, Würzburg, Germany; 6 Institute of Pathology, Ludwig-Maximilians-University München, München, Germany; 7 Department of Musculoskeletal Tissue Regeneration, University of Würzburg, Würzburg, Germany; 8 Department of Internal Medicine I, Medical Center and Faculty of Medicine, University of Freiburg, Breisgau, Germany

**Keywords:** Oncogenes, Cell signalling, Risk factors, Myeloma

## Abstract

Multiple myeloma (MM) is a hematological malignancy whose curability is greatly challenged by recurrent patient relapses and therapy resistance. We have previously proposed the high expression of *ADAM8*, *ADAM9* and *ADAM15* (A Disintegrin And Metalloproteinase 8/9/15) as adverse prognostic markers in MM. This study focused on the so far scarcely researched role of ADAM8/9/15 in MM using two patient cohorts and seven human MM cell lines (HMCL). High *ADAM8*/*9*/*15* expression was associated with high-risk cytogenetic abnormalities and extramedullary disease. Furthermore, *ADAM8*/*15* expression increased with MM progression and in relapsed/refractory MM compared to untreated patient samples. RNA sequencing and gene set enrichment analysis comparing *ADAM8*/*9*/*15*^high/low^ patient samples revealed an upregulation of proliferation markers and proliferation-associated gene sets in *ADAM8*/*9*/*15*^high^ patient samples. High *ADAM8*/*9*/*15* expression correlated with high Ki67 and high *ADAM8*/*15* expression with high MYC protein expression in immunohistochemical stainings of patient tissue. Conversely, siRNA-mediated knockdown of *ADAM8*/*9*/*15* in HMCL downregulated proliferation-related gene sets. Western blotting revealed that *ADAM8* knockdown regulated IGF1R/AKT signaling and *ADAM9* knockdown decreased mTOR activation. Lastly, high *ADAM8*/*9*/*15* expression levels were verified as prognostic markers independent of Ki67/MYC expression and/or high-risk abnormalities. Overall, these findings suggest that ADAM8/9/15 play a role in MM progression and proliferation signaling.

## Introduction

Multiple myeloma (MM) represents approximately 10% of all hematological malignancies and patients present with a variety of clinical manifestations and diverse cytogenetic backgrounds [1]. While MM patient survival has improved due to novel treatments over the last ten years [2], the disease is still generally considered incurable with a median survival of approximately six years [3]. Therapy resistance remains as a major obstacle, causing almost all patients to eventually relapse [1]. Therefore, there is a persisting need to identify novel therapeutic targets and biomarkers associated with disease progression.

The interaction of MM cells with the bone marrow microenvironment, comprised of cellular components (hematopoietic, endothelial and bone marrow stromal cells, osteoblasts, osteocytes, fibroblasts, osteoclasts, etc.) providing growth factors, cytokines and chemokines as well as a non-cellular compartment (extracellular matrix (ECM)), is important for MM cell proliferation and disease progression [4, 5]. We have previously found an association between the high gene expression (GE) of ECM genes such as *A Disintegrin and Metalloproteinase* (*ADAM*) *8*, *ADAM9* and *ADAM15* and a significantly shorter progression-free (PFS) and overall survival (OS) in MM patients [6].

The ADAM family comprises transmembrane and secreted proteins which are involved in various processes important for cancer [7, 8]. ADAM8, 9 and 15 are proteolytically active transmembrane proteins known to shed ectodomains of, among others, growth factors, cytokines and receptors [7, 8]. ADAMs also interact with a variety of other proteins such as integrins and affect integrin signaling pathways such as AKT and mitogen-activated protein kinase (MAPK) signaling [7, 9]. Intracellular signaling is also mediated by their cytoplasmic domains, which contain binding sites for SH3-domain-containing proteins (e.g. phosphoinositide-3-kinase (PI3K)) and amino acid residues which can be phosphorylated by kinases [7, 10].

Accordingly, the upregulation of ADAM8, ADAM9 and ADAM15 has been described, among others, in the context of prognosis, proliferation and progression of e.g. hepatocellular, renal, lung, breast, bladder and colon cancer [9–22].

In contrast, research concerning the role of ADAM8, 9 and 15 in MM is scarce [23–25] and studies thoroughly examining the influence of ADAM8/9/15 on clinical parameters and signaling pathways in MM are lacking. This study therefore aimed to gain insight into the clinical and functional role of ADAM8, ADAM9 and ADAM15 in primary MM and human MM cell lines (HMCL).

## Materials and methods

### Cell culture

The HMCL L-363, JJN-3, KMS-12-BM, U-266 and AMO-1 were purchased from the “Deutsche Sammlung von Mikroorganismen und Zellkulturen GmbH” (DSMZ, Braunschweig, Germany), MM.1S from LGC Biolabs (Wesel, Germany) and KMS-11 was acquired from the Japanese Collection of Research Bioresources Cell Bank (JCRB1179).

All cell lines were cultured in RPMI-1640 medium (Thermo Fisher Scientific, Waltham, MA, USA) supplemented with 10% fetal bovine serum (FBS), 2 mM L-glutamine and 1 mM sodium pyruvate (all from PAN-Biotech, Aidenbach, Germany) at 37 °C and 5% CO_2_ for a maximum of 3 months and regularly tested for mycoplasma (VenorGEM One-Step kit (Minerva Biolabs, Berlin, Germany)). Cell lines were authenticated using the short tandem repeats profiling.

### siRNA-mediated knockdown

HMCL were kept in culturing medium supplemented with 15% FBS for one night before transfection. 6 × 10^6^ cells per condition were washed in PBS and resuspended in 200 µl unsupplemented RPMI-1640 medium containing 2.5 µM scrambled siRNA (scr-siRNA) (All Stars Negative Control siRNA, QIAGEN, Hilden, Germany, SI03650318) or *ADAM8*-, *ADAM9*- or *ADAM15*-specific siRNA (Supplementary Tables S1 and S2) immediately before electroporation in 2 mm cuvettes using the Gene Pulser Xcell electroporation system (BIO-RAD, Hercules, CA, USA) with an exponential program (capacitance: 980 µF) at 180 V (AMO-1, JJN-3, L-363), 200 V (MM.1S, KMS-11, KMS-12-BM) or 230 V (U-266) [26]. 200 µl unsupplemented medium were added to the cuvettes immediately after electroporation, the cells transferred into tubes containing further medium and incubated for 5 min before being transferred into 6-well plates containing 7 ml of prewarmed electroporation medium (EP medium) (RPMI supplemented with 15% FBS, 1% P/S, 2 mM L-glutamine). After 24 h, density gradient centrifugation was performed to separate living cells from dead cells. The cells were centrifuged, resuspended in 2.5 ml EP-medium mixed with 750 µl OptiPrep (Serumwerk, Bernburg, Germany)) and the suspension overlaid with 200 µl PBS. After centrifugation at 3500 rpm for 7 min, the viable cells were transferred from the medium-PBS interface into fresh EP-medium, centrifuged at 1000 rpm for 3 min, resuspended in a suitable amount of EP-medium and transferred to fresh 6-well plates to be incubated for another 24 h. 48 h after electroporation, cells were pelleted and lysates or RNA prepared for Western blotting or RNA sequencing, respectively. SiRNA knockdowns were performed at least three times in independent experiments for the assessment with Western blot. RNA was only extracted from one experiment.

### Western blotting

Protein extraction, SDS-PAGE and Western blotting were performed as previously described [27]. 20 µg protein were loaded. Antibodies are listed in Supplementary Table S1. Band intensities were evaluated using the “gels” tool in Image J. Intensities for each marker were normalized to the corresponding GAPDH signal detected on the same blot. For siRNA knockdown experiments, the normalized expression of the siRNA samples was subsequently normalized to the respective scr-siRNA control sample. Western blot quantifications were statistically evaluated using the two-tailed t-test.

### Patient samples

RNA sequencing of 73 previously CD138-sorted samples from 51 patients (“validation cohort”) was performed. For patient characteristics and corresponding experimental data see Supplementary Table S3. The study was approved by the Ethics Committee of the Medical Faculty, University of Würzburg (reference numbers 76/13 and 149/23-am). All methods were performed in accordance with the relevant guidelines and regulations. Informed consent was obtained from all subjects.

### RNA sequencing

#### RNA extraction and sequencing

One RNA sequencing dataset was available from the Multiple Myeloma Research Foundation (MMRF CoMMpass study; 921 samples from 806 patients).

RNA from primary samples of the validation cohort was extracted using the DNA/RNA Micro kit (QIAGEN #80284) according to the manufacturers’ instructions. Following mRNA library preparation (Illumina), 100 bp paired end sequencing was performed on a NovaSeq (Illumina). Mapping was performed with STAR v2.7.2b and counts generated with featureCounts v1.6.4. Non-integer counts were rounded in R prior to analysis with DESeq2.

RNA from siRNA knockdown experiments was extracted from approximately 5 × 10^5^ cells using the RNeasy mini kit (QIAGEN #74104), followed by Illumina mRNA library preparation. 150 bp paired end sequencing using a minimum of 200 ng total RNA were performed on a NovaSeq. Reads were mapped with Hisat2 v2.0.5 and counts generated with featureCounts v1.5.0-p3.

Sequencing quality parameters are summarized in Supplementary Table S4. Raw data has been uploaded to the European Genome-Phenome Archive (Accession: EGAS50000000392).

#### Analysis of differentially expressed genes

Differentially expressed genes between primary MM samples with the highest or lowest GE of *ADAM8*/*9*/*15* (*n* = 92/condition in the MMRF cohort; *n* = 18/condition in the validation cohort) or before and after siRNA knockdown of *ADAM8*/*9*/*15* in HMCL (*n* = 5–7/condition) were detected using DESeq2 in R [28]. For more details and information concerning sample grouping see supplementary methods. Principal component analysis (PCA) was performed on the vst-transformed DESeq datasets using the R packages DESeq2 [28], magrittr [29] and ggplot2 [30]. Volcano plots were created using EnhancedVolcano [31].

#### Gene set enrichment analysis (GSEA)

GSEA (GSEA 4.3.2, Broad Institute [32]) was performed on normalized counts matrices obtained from DESeq2 using hallmark gene sets (h.all.v2023.1.Hs.symbols.gmt) with a weighted enrichment statistic. FDR *q* values < 0.25 were considered significant.

### Statistical comparison of *ADAM8*/*9*/*15* gene expression between samples with different cytogenetic backgrounds and clinical parameters

*ADAM8*/*9*/*15* GE (TPM) was compared between samples with or without high-risk cytogenetic abnormalities using the Mann–Whitney U test in both cohorts. *P* values were adjusted using the Benjamini–Hochberg correction in R.

In the MMRF cohort, *ADAM8*/*9*/*15* GE (TPM) was additionally compared between the baseline sample and sample(s) taken from the same patient at progressive disease using the Wilcoxon-test (*n* = 59 patients). When more than one progressive disease sample was available for a patient, samples were treated as replicates.

In the validation cohort, *ADAM8*/*9*/*15* GE (TPM) was compared between samples from patients with (*n* = 9) or without (*n* = 41) extramedullary disease (EMD) at the time of biopsy and between samples obtained from untreated patients (*n* = 13) and relapsed/refractory MM (RRMM) (*n* = 34) using the Mann–Whitney-U test. Where more than one sample was available from a patient, the mean *ADAM8*/*9*/*15* GE of all samples was used if all samples belonged to the same group.

### Immunohistochemical staining and analysis

MYC was previously stained on formalin-fixed paraffin-embedded (FFPE) bone marrow material from samples within the validation cohort using immunohistochemistry (IHC) [33]. Ki67 (MIB-1, Dako, 1:800) and CD138 (MI15, Dako, 1:100) were stained on consecutive slides after boiling in citric acid (pH 6.0).

The percentage of Ki67-positive CD138-positive tumor cells was evaluated by an expert hematopathologist (AR). Enrichment of Ki67^high^ (≥30% Ki67^+^ CD138^+^ cells) or MYC^high^ (≥40% MYC^+^ CD138^+^ cells) samples in the *ADAM8*/*9*/*15*^high/low^ (GE >/≤ mean) groups was investigated using Fisher’s exact test. MYC (% MYC^+^ CD138^+^ cells) and Ki67 expression (% Ki67^+^ CD138^+^ cells) was additionally compared between *ADAM8*/*9*/*15*^high/low^ samples using the Mann–Whitney-U test.

### Survival analyses

High GE of *ADAM8*, *ADAM9* and *ADAM15* was correlated with OS and PFS using the Kaplan-Meier method and log rank test in the validation cohort. Patients were grouped as *ADAM8*/*9*/*15*^high^ when the median GE of all samples from the respective patient was higher than the mean GE (TPM) of all samples in the cohort.

Multivariate survival analysis was performed using the Cox proportional hazards model with high/low MYC, Ki67 and *ADAM8*/*9*/*15* expression or high-risk cytogenetic abnormalities and *ADAM8*/*9*/*15* expression as variables. Patients were MYC^high^ or Ki67^high^ when at least one sample had ≥40% MYC-positive or ≥30% Ki67-positive MM cells (measured by IHC). A cytogenetic abnormality was considered to be present in a patient when it was detected in at least one sample. Analyses were performed using GraphPad Prism 9.

## Results

### High expression of *ADAM8*, *ADAM9* and *ADAM15* is associated with high-risk cytogenetic abnormalities

Looking at clinically relevant molecular parameters, we compared the GE of *ADAM8*/*9*/*15* between patient samples with or without high-risk cytogenetic abnormalities (defined in ref. [1]) in two patient cohorts (MMRF, in-house validation). The 1q gain/amplification was associated with a significantly higher GE of *ADAM8*, *ADAM9* and *ADAM15* in the MMRF cohort (Table 1). Additionally, *ADAM8* GE was significantly higher in samples with *TP53* abnormalities or del17p in the MMRF cohort or validation cohort, respectively (Table 1). *ADAM9* GE was higher in samples with the translocation t(14;16) in the MMRF cohort (Table 1). A significant but inconsistent association between *ADAM8* and *ADAM9* GE levels and the presence of t(4;14) was also observed in the MMRF cohort (Table 1).

**Table 1:** Comparison of *ADAM8*, *ADAM9* and *ADAM15* (GE) (TPM) between samples with and without cytogenetic abnormalities associated with a high risk status (defined in ref. [1]).

Cytogenetic abnormality absent vs. present							
MMRF cohort							
Abnormality	*n* =	*ADAM8* GE	padj.	*ADAM9* GE	padj.	*ADAM15* GE	padj.
del17p	428 vs. 58	2.78 vs. 3.82	0.105	4.95 vs. 6.50	0.285	10.69 vs. 9.841	0.687
TP53	435 vs. 88	2.74 vs. 4.06	**0.024**	4.84 vs. 6.32	0.067	10.47 vs. 11.18	0.296
1q gain/amp	352 vs. 222	2.72 vs. 3.49	**0.024**	4.21 vs. 6.69	**1.31E−07**	8.56 vs. 13.14	**1.80E−13**
t(4;14)	521 vs. 119	3.35 vs. 2.00	**0.013**	4.79 vs. 6.31	**0.013**	10.25 vs. 10.60	0.687
t(14;16)	539 vs. 57	3.02 vs. 3.82	0.285	4.84 vs. 6.78	**0.031**	10.08 vs. 12.00	0.077
t(14;20)	357 vs. 10	2.53 vs. 3.10	0.767	4.93 vs. 6.13	0.785	10.21 vs. 11.15	0.767
**Validation cohort**							
**Abnormality**	***n*** =	***ADAM8*** **GE**	**padj**.	***ADAM9*** **GE**	**padj**.	***ADAM15*** **GE**	**padj**.
del17p	43 vs. 18	2.22 vs. 7.01	**0.015**	2.69 vs. 3.38	0.477	8.67 vs. 11.84	0.463
TP53_mut	50 vs. 10	2.93 vs. 3.99	0.563	2.35 vs. 4.82	0.170	9.83 vs. 14.88	0.284
1q gain/amp	34 vs. 21	2.75 vs. 3.49	0.765	2.41 vs. 3.29	0.765	10.03 vs. 7.15	0.912
t(4;14)	42 vs. 18	2.67 vs. 4.33	0.463	2.69 vs. 1.90	0.981	9.83 vs. 7.09	0.942
t(14;16)	54 vs. 2	2.83 vs. 6.41	0.563	2.67 vs. 5.28	0.563	9.19 vs. 49.17	0.284

Statistical test was Mann–Whitney-U. *P* values were adjusted (padj.) for testing of multiple hypotheses using the Benjamini–Hochberg procedure in R. Translocation status for t(14;20) was not available in the validation cohort. Amp: amplification.

In summary, supporting a clinical relevance, the presence of at least one high-risk cytogenetic abnormality was associated with a higher GE of *ADAM8*/*9*/*15* in the MMRF and/or validation cohort.

Adding to our previous findings, where a high GE of *ADAM8*, *ADAM9* and *ADAM15* was associated with shorter PFS and OS in the MMRF cohort [6], multivariate survival analyses considering *ADAM8*/*9*/*15* GE and the respective cytogenetic abnormalities which were associated with a high *ADAM8*/*9*/*15* GE (Table 1) revealed high *ADAM8*/*9*/*15* GE as independent prognostic markers for shorter PFS and OS in the MMRF cohort (Supplementary Table S5).

Furthermore, high GE of *ADAM8*, *ADAM9* and *ADAM15* was also associated with a significantly shorter survival in the newly sequenced validation cohort (Supplementary Fig. S1) and multivariate survival analysis verified high *ADAM8* GE as prognostic for shorter OS and PFS independent of the presence of del17p (Supplementary Table S6).

### *ADAM8*, *ADAM9* and *ADAM15* upregulation is associated with progressive disease in MM

**Fig. 1:**
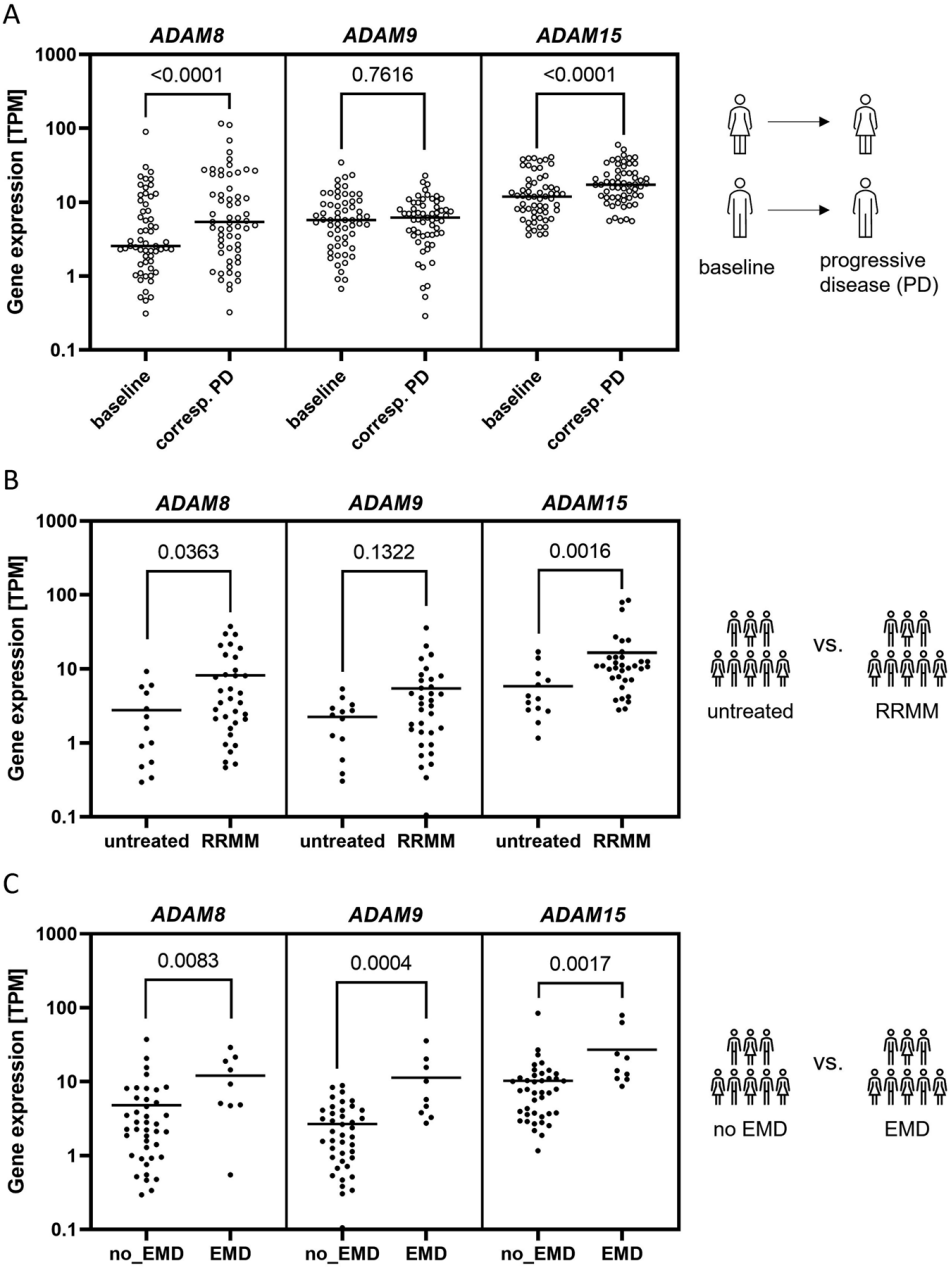
High expression of *ADAM8*, *ADAM9* and *ADAM15* is associated with progressive disease. **A** Comparison of *ADAM8*, *ADAM9* and *ADAM15* GE between the baseline sample (first sample acquired when patient entered the study) and corresponding samples taken from the same patient at a stage of progressive disease (corresp. PD) in the MMRF cohort (*n* = 59 patients in analysis). Samples were treated as replicates if more than one PD sample was available for a patient. Statistical test was Wilcoxon-test. Lines show the mean GE. **B** Comparison of *ADAM8*, *ADAM9* and *ADAM15* GE between unpaired samples (except for 2) obtained from untreated patients (*n* = 13) and RRMM (*n* = 34) from the validation cohort. When more than one sample taken at the same stage was available for a patient, the mean was used. Statistical test was Mann–Whitney-U. Lines show the mean GE. **C** Comparison of *ADAM8*, *ADAM9* and *ADAM15* GE between unpaired samples from patients with or without extramedullary disease (EMD: *n* = 9 or no_EMD: *n* = 41) at biopsy in the validation cohort. Where more than one sample with the same EMD status was available from one patient, the mean GE of these samples was used. One patient acquired EMD within the course of the study, the remaining patients did not change groups. Statistical test was Mann–Whitney-U. Lines show the mean GE. Patient information and treatment for the validation cohort is summarized in Supplementary Table S3.

Next, we assessed the role of *ADAM8*, *ADAM9* and *ADAM15* GE in the context of MM progression in both patient cohorts. Analysis of paired samples revealed a significant upregulation of *ADAM8* and *ADAM15* GE in samples obtained at a stage of progressive disease compared to the corresponding baseline sample collected from the same patient in the MMRF cohort (Fig. 1A). *ADAM8* and *ADAM15* GE was also significantly higher in RRMM samples compared to samples from untreated patients in the validation cohort (Fig. 1B). Additionally, MM samples from patients with EMD at the time of biopsy had a significantly higher *ADAM8*, *ADAM9* and *ADAM15* GE than samples from patients with no EMD in the validation cohort (Fig. 1C).

In summary, high GE of *ADAM8*, *ADAM9* and *ADAM15* was verified as prognostic for shorter patient survival and associated with high-risk cytogenetics as well as disease progression.

### *ADAM8*/*9*/*15* influence proliferation and survival signaling in MM

**Fig. 2:**
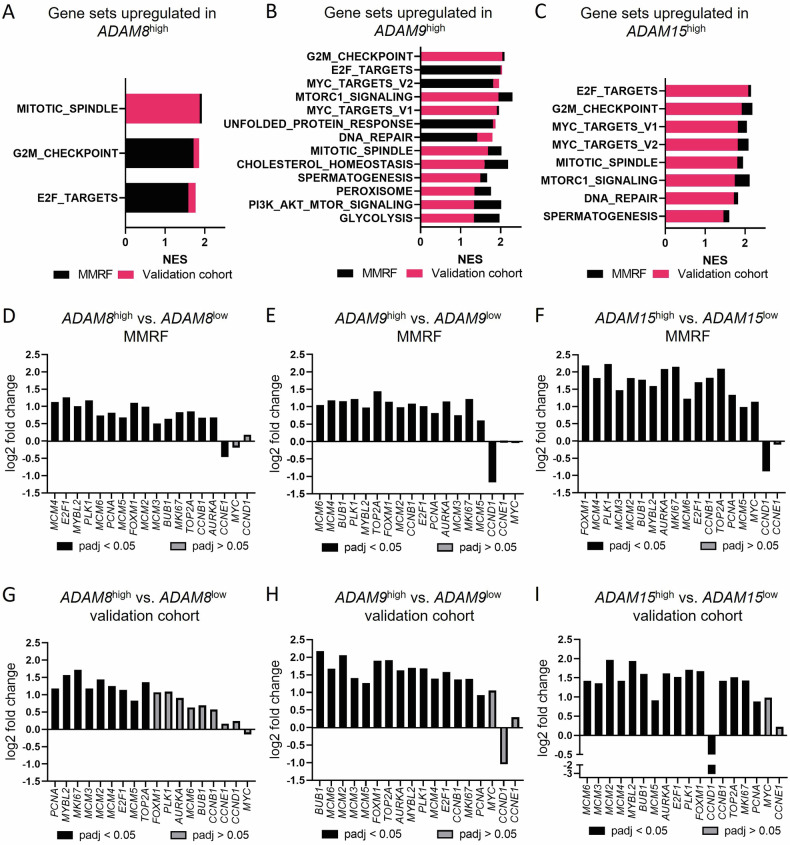
*ADAM8*/*9*/*15* expression levels influence proliferation signaling in MM. Summary of gene sets where a significant enrichment (FDR *q* value < 0.25) was found in **A** *ADAM8*^high^, **B** *ADAM9*^high^ or **C** *ADAM15*^high^ patient samples from both the MMRF (black) and validation cohort (red). NES Normalized enrichment score. A summary of all enriched gene sets is shown in Supplementary Table S8. **D**–**I** Log2 fold change of expression of commonly used proliferation markers between *ADAM8*/*9*/*15*^high^ vs. *ADAM8*/*9*/*15*^low^ primary MM samples. Top 10% (MMRF (**D**–**F**)) or 25% (validation cohort (**G**–**I**)) of samples with the highest/lowest *ADAM8*/*9*/*15* GE were included. padj values (*p* value adjusted for multiple hypothesis testing) increase from left to right. Significantly differentially expressed genes (padj < 0.05) have black bars, genes with padj > 0.05 are depicted in gray. A summary of all differentially expressed genes is shown in Supplementary Table S7.

To gain insight into which signaling pathways may be regulated by ADAM8/9/15 in MM, we compared the GE profiles of *ADAM8*/*9*/*15*^high^ and *ADAM8*/*9*/*15*^low^ patient samples from both the MMRF and validation cohort using GSEA. For PCA and volcano plots of RNA sequencing data see Supplementary Figs. S2–S4. Differentially expressed genes are summarized in Supplementary Table S7. There was a considerable overlap of significantly enriched gene sets (FDR *q* value < 0.25) between the two patient cohorts and between the analyses for the different ADAM genes (Fig. 2A–C). The vast majority of these gene sets were associated with cell cycle, proliferation and survival signaling (Fig. 2A–C). More precisely, the gene sets “G2/M checkpoint”, “E2F targets” and “mitotic spindle” were significantly enriched in *ADAM8*/*9*/*15*^high^ samples from both patient cohorts (Fig. 2A–C) and “DNA repair”, “MYC targets” and “MTORC1 signaling” were enriched in *ADAM9*/*15*^high^ samples from both cohorts (Fig. 2B, C). For a summary of all enriched gene sets in the individual cohorts see Supplementary Table S8.

We subsequently assessed the differential expression of common proliferation markers [34, 35] between *ADAM8*/*9*/*15*^high^ and *ADAM8*/*9*/*15*^low^ patient samples. Virtually all of the significantly differentially expressed (padj<0.05) proliferation markers investigated were upregulated in the *ADAM8*/*9*/*15*^high^ samples from both patient cohorts (Fig. 2D–I). The only exceptions were the significant downregulation of *Cyclin E1* (*CCNE1*) in *ADAM8*^high^ samples from the MMRF cohort and of *Cyclin D1* (*CCND1*) in *ADAM9*^high^ samples from the MMRF cohort and *ADAM15*^high^ samples from both cohorts (Fig. 2D, E, F, I).

Since *CCND1* upregulation can be caused by the translocation t(11;14) [36], we assessed whether there was an enrichment of samples with t(11;14) in the *ADAM9/15*^low^ samples. Samples with t(11;14) were significantly enriched in the *ADAM9*^low^ samples from the MMRF but not the *ADAM15*^low^ samples from either of the cohorts (Supplementary Fig. S5).

In summary, high *ADAM8*/*9*/*15* expression levels influenced important proliferation and survival signaling pathways in MM patients from two different cohorts.

### High *ADAM8*/*9*/*15* expression correlates with high Ki67 and MYC protein expression

**Fig. 3:**
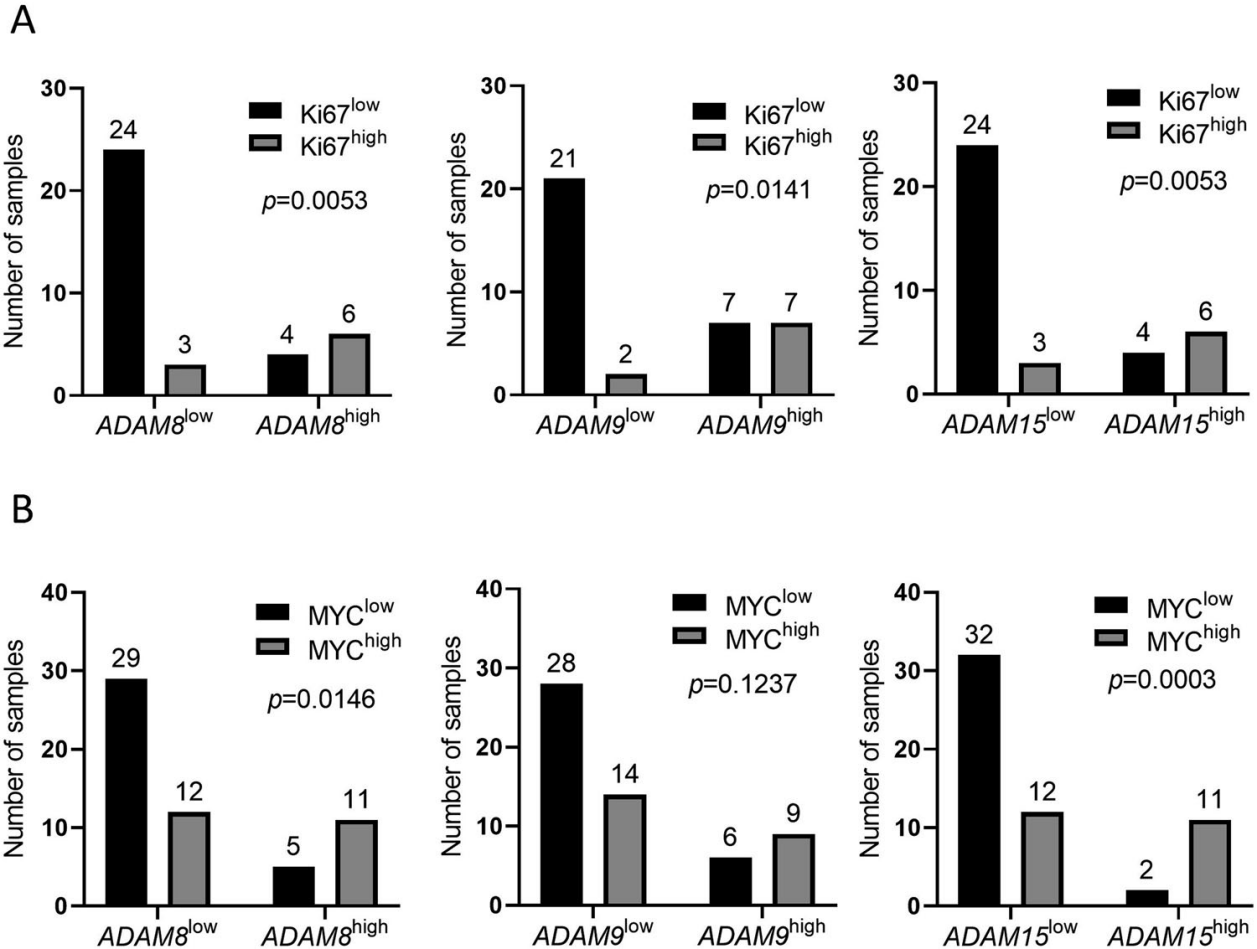
Ki67 and MYC protein expression in samples from the validation cohort. **A** Samples with high Ki67 protein expression determined by IHC are significantly enriched in *ADAM8*/*9*/*15*^high^ patient samples from the validation cohort. **B** Samples with high MYC protein expression determined by IHC are significantly enriched in *ADAM8*/*15*^high^ but not in *ADAM9*^high^ patient samples from the validation cohort. *ADAM8*/*9*/*15*^high/low^: *ADAM8*/*9*/*15* GE>/≤ mean of all samples. Ki67^high/low^: ≥30%/<30% Ki^+^ CD138^+^ cells. MYC^high/low^: ≥40%/<40% MYC^+^ CD138^+^ cells. Statistical test was Fisher’s exact test. For exemplary Ki67 stainings see Supplementary Fig. S7. For MYC stainings see ref. [33].

Since the high expression of *ADAM8*/*9*/*15* was associated with an upregulation of proliferation marker gene expression (Fig. 2), we subsequently assessed Ki67 and MYC protein expression detected by IHC in *ADAM8*/*9*/*15*^high^ and *ADAM8*/*9*/*15*^low^ patient samples from the validation cohort (Fig. 3, Supplementary Figs. S6, S7). We found a significant association between high *ADAM8*/*9*/*15* and high Ki67 expression (Fig. 3A, Supplementary Fig. S6). Moreover, high *ADAM8* and *ADAM15* expression were associated with high MYC expression levels (Fig. 3B, Supplementary Fig. S6).

**Fig. 4:**
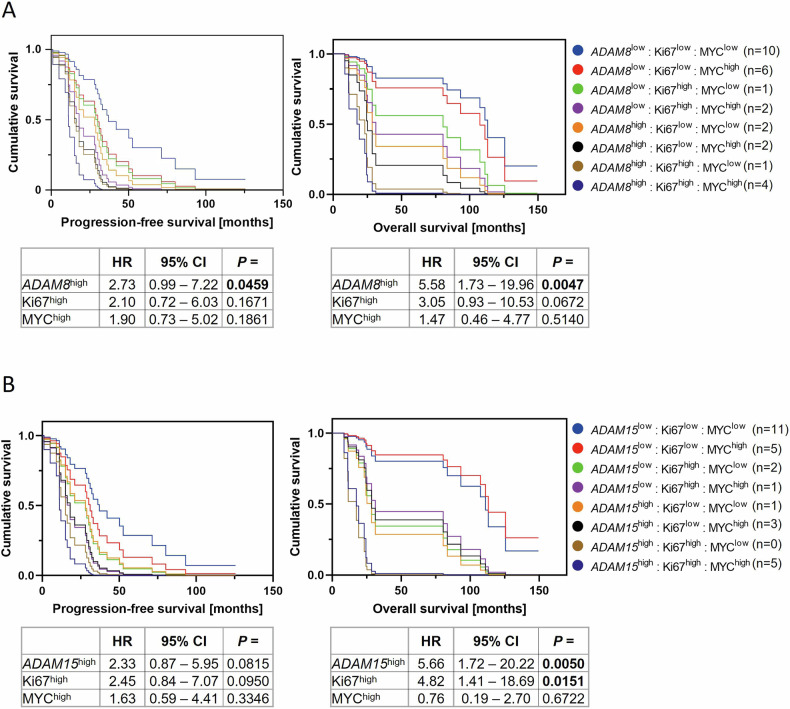
High *ADAM8* and *ADAM15* expression levels are independent prognostic markers in MM. Cox proportional hazards model assessing the effect of high Ki67, MYC and (**A**) *ADAM8* or (**B**) *ADAM15* expression on progression-free survival (left) and overall survival (right) in the validation cohort. *ADAM8*/*15*^high^ patients have an *ADAM8*/*15* GE >mean of all samples. Ki67^high^ patients had at least one sample with ≥30% Ki67-expressing CD138^+^ cells. MYC^high^ was assigned to a patient if at least one sample contained ≥40% MYC-expressing MM cells. *n* = 28 patients. HR hazard ratio, CI confidence interval.

Since high Ki67 and MYC protein expression have been shown to correlate with shorter PFS and/or OS in MM [33, 37–39], the prognostic value of high *ADAM8*/*9*/*15* GE was subsequently reassessed in multivariate survival analyses. Cox regression considering Ki67 and MYC protein and *ADAM8* GE confirmed high *ADAM8* GE as an independent prognostic marker for shorter PFS and OS (Fig. 4A).

Cox regression considering both *ADAM9* and Ki67 expression only verified high Ki67 expression as an independent prognostic marker for shorter PFS and OS (Supplementary Fig. S8).

Multivariate survival analyses considering *ADAM15*, Ki67 and MYC expression confirmed both the high *ADAM15* and Ki67 expression as independent predictors for worse OS but not for PFS (Fig. 4B). For Cox regressions considering only *ADAM8*/*15* GE and either Ki67 or MYC expression see Supplementary Figs. S9, S10.

### siRNA knockdown of *ADAM8*/*9*/*15* influences proliferation/survival signaling in HMCL

In an experimental approach using siRNA knockdowns of *ADAM8*, *ADAM9* or *ADAM15* in HMCL, we aimed to verify the influence of ADAM8/9/15 expression levels on the signaling pathways enriched in the GSEA comparing the expression profiles of *ADAM8*/*9*/*15*^high/low^ patient samples.

ADAM8/9/15 protein expression was assessed prior to siRNA knockdowns in seven HMCL by Western blotting. ADAM8 and ADAM15 were expressed in 5/7 HMCL and ADAM9 was expressed in all seven HMCL (Supplementary Fig. S11). Knockdowns were only performed in the HMCL with considerable expression of the respective ADAMs.

Similar to what was observed in the two patient cohorts, GSEA comparing the gene expression profiles of HMCL before (scr-siRNA control) and after siRNA knockdown of *ADAM8*/*9*/*15* revealed a significant enrichment of gene sets associated with proliferation, cell cycle and survival (Supplementary Fig. S12, Supplementary Table S8).

Moreover, we assessed the effect of *ADAM8*/*9*/*15* siRNA knockdown on further signaling pathways of known importance in MM [39–42] which were highly enriched in the GSEA of patient samples using Western blots.

**Fig. 5:**
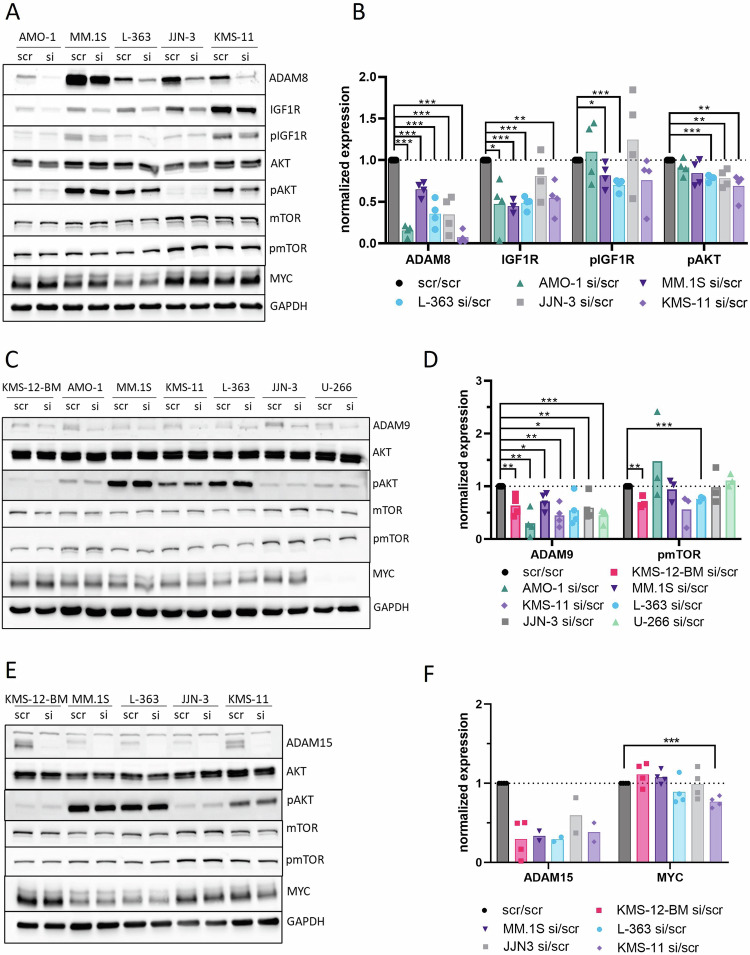
*ADAM8*/*9*/*15* siRNA knockdowns in HMCL. **A**–**F** Evaluation of expression and activation of members of the PI3K/AKT/mTOR signaling pathway and MYC expression using Western blotting after *ADAM8*/*9*/*15* siRNA knockdown. Cells were transfected with either scr-siRNA control (scr) or *ADAM8*/*9*/*15*-specific siRNA (si) in four independent rounds of experiments, respectively. **A**, **C**, **E** Representative Western blots. Pan and phospho-markers were detected on separate blots and only the relevant area of the blots is shown. Housekeeper (GAPDH) was detected on each blot, representative GAPDH staining is shown. **B**, **D**, **F** Summary of normalized expression for all evaluable rounds of experiments for markers where an effect was observed. The expression of each marker was first normalized to the expression of the housekeeper and subsequently the siRNA samples were normalized to the scr-siRNA controls. Each data point represents one independent round of experiments. Bars show the mean. Statistical test was two-tailed t-test. * for *p* < 0.05, ** for *p* < 0.01, *** for *p* < 0.001. **B** *n* = 4 for all markers statistically evaluated except for IGF1R in MM.1S (*n* = 3). **D** *n* = 4 for ADAM9, *n* = 3 for pmTOR. **F** *n* = 4 for MYC. ADAM15 was only evaluable for all cell lines in two rounds of experiments because of a complete lack of bands for the siRNA transfected HMCLs in the other rounds due to a complete knockdown (see blot in **E**). The difference in ADAM15 expression levels between scr and si was therefore not statistically evaluated.

“PI3K/AKT/MTOR signaling” was the top enriched gene set (highest normalized enrichment score) in the *ADAM8*^high^ patient samples from the MMRF cohort (Supplementary Table S8). *ADAM8* knockdown significantly reduced the expression of the receptor tyrosine kinase insulin-like growth factor 1 receptor (IGF1R), which is known to regulate this pathway [43], by approximately half in 4/5 HMCL (Fig. 5A, B). Moreover, pIGF1R levels were clearly reduced in 3/5 HMCL (Fig. 5A, B). AKT expression was unaffected while pAKT was significantly reduced in 3/5 HMCL (Fig. 5A, B). A conclusive effect on mTOR expression or activation was not observed (Fig. 5A, B).

“MTORC1 signaling” was the top enriched gene set in *ADAM9*^high^ patient samples from the MMRF cohort (Supplementary Table S8) and “MYC targets” were enriched in *ADAM9*^high^ patient samples from both cohorts as well as in the comparison of HMCL before and after *ADAM9* siRNA knockdown (Fig. 2B, Supplementary Fig. S12). AKT, pAKT, mTOR or MYC expression were not affected by *ADAM9* siRNA knockdown on protein level. However, pmTOR was significantly reduced in KMS-12-BM and L-363 and also clearly reduced by 44% in KMS-11 (Fig. 5C, D).

The gene set “MTORC1 signaling” was enriched in *ADAM15*^high^ samples from both cohorts and in the comparison of HMCL before and after *ADAM15* siRNA knockdown (Fig. 2C, Supplementary Fig. S12). “MYC targets” were enriched in *ADAM15*^high^ samples from both patient cohorts (Fig. 2C). A conclusive effect of *ADAM15* siRNA knockdown on the mTOR signaling members assessed herein was not observed in Western blots. However, MYC was significantly downregulated in KMS-11 (Fig. 5E, F).

In summary, similar to our observations in the two patient cohorts, ADAM8/9/15 expression levels influenced survival and proliferation signaling pathways in an experimental approach using siRNA knockdowns in HMCL.

## Discussion

Our study validates the previously proposed [6] prognostic potential of a high *ADAM8*, *ADAM9* and *ADAM15* GE in a newly sequenced patient cohort and investigates the thus far scarcely studied clinical and functional role of ADAM8, ADAM9 and ADAM15 in two MM patient cohorts (MMRF CoMMpass study cohort and our own validation cohort) and seven HMCL.

Focusing on clinical aspects, we found an association between the presence of high-risk cytogenetic abnormalities and an increased *ADAM8*/*9*/*15* GE. Remarkably, the 1q amplification/gain was associated with a higher GE of all three ADAMs assessed herein. This association was particularly strong for *ADAM15*, which is in line with the fact that *ADAM15* is encoded on chromosome 1q21.3. Since high-risk cytogenetic abnormalities are associated with shorter patient survival and e.g. the incidence of 1q amplifications/gains increases with MM progression [44–47], the role of *ADAM8*/*9*/*15* in the prognosis and progression of MM was investigated in more detail.

Underlining the clinical relevance of ADAM8/9/15, multivariate survival analyses verified high *ADAM8*/*9*/*15* expression levels as robust prognostic markers for shorter patient survival independent of the association with high-risk cytogenetics in this study.

Apart from high-risk cytogenetic abnormalities, high *ADAM8* and *ADAM15* expression also correlated with high MYC protein expression and high *ADAM8*, *ADAM9* and *ADAM15* expression correlated with high Ki67 protein expression in MM patients in the current study. High Ki67 expression is a known prognostic marker in MM [37, 38]. Moreover, MM is generally considered to be MYC-driven [41] and high MYC expression is known to affect OS [33, 39] and to correlate with a high proliferation index (Ki67) [39]. Nevertheless, high expression levels of *ADAM8* and *ADAM15* were also verified as prognostic markers independent of Ki67 and MYC expression levels in multivariate survival analyses, underlining their potential suitability as biomarkers.

Next, we found an upregulation of *ADAM8*, *ADAM9* and *ADAM15* in patients with EMD, which is associated with aggressive and progressive disease [48], in the validation cohort. Similarly, *ADAM8* and *ADAM15* GE also increased with disease progression in the MMRF cohort and *ADAM8* and *ADAM15* GE increased in RRMM compared to untreated samples from the validation cohort. These results imply a possible role for *ADAM8/9/15* in MM progression, relapse and therapy resistance. Accordingly, expression levels of ADAM8, ADAM9 and ADAM15 have all been shown to be involved in the progression and/or metastasis/invasion of various solid cancers [9, 11–13, 15–18, 20, 21]. Furthermore, ADAM8 has been linked to chemoresistance in solid cancers [9] and tyrosine kinase inhibitor therapy resistance in chronic myeloid leukemia cells [49].

GSEA comparing *ADAM8*/*9*/*15*^high/low^ MM patient samples of two different cohorts or HMCL before and after *ADAM8*/*9*/*15* siRNA knockdown revealed an upregulation of gene sets associated with proliferation and cell cycle/growth in the *ADAM8*/*9*/*15*^high^ groups, with “G2/M checkpoint”, “E2F targets”, “MYC targets” and “MTORC1 signaling” among the most frequently upregulated gene sets. The considerable overlap of gene sets that were enriched when comparing *ADAM8*/*9*/*15*^high/low^ patient samples from the two different cohorts and when comparing HMCL before and after active downregulation of ADAM8/9/15 by siRNA knockdown supports the reliability of these results.

Interestingly, it has been shown that triple-relapsed MM compared to newly diagnosed or relapsed samples pre-daratumumab exposure [50], and extramedullary MM compared to newly diagnosed MM [51] upregulate E2F and MYC targets as well as the G2/M checkpoint gene set, further underscoring the association between *ADAM8*/*9*/*15* upregulation and disease progression.

Consistent with the GSEA results, which suggested an influence of *ADAM8*/*9*/*15* expression on proliferation signaling, almost all proliferation markers [34, 35] were upregulated in *ADAM8*/*9*/*15*^high^ samples on RNA level in both patient cohorts. The only exceptions were the downregulation of *CCND1* in *ADAM9*/*15*^high^, *CCNE1* in the *ADAM8*^high^ samples in the MMRF and/or the validation cohort. *CCND1* is a known player in MM due to the common translocation t(11;14) [36]. The enrichment of cases with t(11;14) in the *ADAM9*^low^ samples from the MMRF cohort might explain why *CCND1* appeared to be downregulated in the *ADAM9*^high^ samples. However, samples with t(11;14) were evenly distributed between *ADAM15*^high/low^ samples from both cohorts, and the downregulation of *CCND1* is therefore most likely explained by the cyclic up- and downregulation of cyclins depending on the cell cycle phase [52]. In line with the enrichment of the G2/M checkpoint gene set, *CCNB1*, important for the G2/M transition [52], was upregulated where *CCND1* or *CCNE1*, important factors in G1 and S phase [52], were downregulated.

*MKI67* (encoding Ki67), one of the most well-known proliferation markers, which is used in routine tumor diagnostics to assess the proliferation index, was significantly upregulated in *ADAM8*/*9*/*15*^high^ samples from both cohorts on RNA level and high *ADAM8*/*9*/*15* expression also correlated with high Ki67 protein expression, further strengthening the hypothesis that high *ADAM8*, *ADAM9* and *ADAM15* expression may be associated with increased proliferation.

In line with that, this study found evidence that ADAM8 might influence the PI3K/AKT/mTOR signaling pathway, as this pathway was enriched the most in *ADAM8*^high^ patient samples and IGF1R expression and activation as well as AKT activation was downregulated by *ADAM8* siRNA knockdown in HMCL. The IGF1R/PI3K/AKT/mTOR signaling cascade is commonly activated in MM and regulates MM cell growth, proliferation and survival [40, 53–55]. Furthermore, an influence of ADAM8 expression on MAPK and AKT signaling has also been observed in other cancers [11, 12], underscoring our current findings in MM.

Genes associated with MTORC1 signaling showed a high enrichment in *ADAM9*/*15*^high^ compared to *ADAM9*/*15*^low^ patient samples. Studies have described mTOR as an important factor in MM, influencing e.g. proliferation, growth, survival, invasion and chemoresistance [40]. In line with the GSEA results, *ADAM9* siRNA knockdown reduced mTOR activation in several HMCL. While pAKT was largely unaffected, this could be explained by alternative kinases phosphorylating mTOR [56]. No conclusive effect on the members of the MTORC1 signaling pathway assessed herein was observed upon *ADAM15* siRNA knockdown. However, since MTORC1 activity can be influenced by a number of factors (phosphorylation by various kinases, interacting partners in the complex, nutrient supply, localization [57]), conclusively verifying the effect of ADAM9/15 expression on MTORC1 signaling is beyond the scope of this project. Nevertheless, these data still provide further evidence that ADAM9 may influence MTORC1 signaling in MM, as has been described for colorectal cancer cells [58].

Consistent with the influence on proliferation signaling in MM observed herein, ADAM8, 9 and 15 have been shown to influence the proliferation of e.g. hepatocellular, renal and prostate cancer [12, 13, 19, 22].

Considering the various functions of ADAMs, possible mechanisms by which ADAM8/9/15 expression levels might influence proliferation signaling could be e.g. the cleavage of growth factors and receptors or the interaction with integrins [7–9]. For instance, it has been shown that ADAM9, expressed on MM cells, can interact with integrin αvβ5 on osteoblasts and thereby promote their production of interleukin-6 [24] which, in turn, can stimulate the proliferation of MM cells and prevent apoptosis [59]. Nevertheless, the exact mechanisms by which ADAM8/9/15 influence proliferation signaling in MM remain to be elucidated in future studies.

In conclusion, this study showed that high expression levels of *ADAM8*, *ADAM9* and *ADAM15* are interesting biomarkers of prognostic relevance in MM, that are linked to a high-risk status and disease progression and influence several important survival and proliferation signaling pathways. Further studies should therefore assess the potential usefulness of targeting these ADAMs therapeutically.

## Supplementary information

Supplementary methods, figures and tables S1, S5, S6

Supplementary Table S2

Supplementary Table S3

Supplementary Table S4

Supplementary Table S7

Supplementary Table S8

## Data Availability

The datasets generated and/or analyzed during the current study have been uploaded to the European Genome-Phenome Archive (EGA) (Accession: EGAS50000000392).
