# Supplementary material for: Association of ADAM family members with proliferation signaling and disease progression in multiple myeloma
Source: Blood Cancer J. 2024 Sep 11;14(1):156. doi: 10.1038/s41408-024-01133-4 (PMC11390935; doi:10.1038/s41408-024-01133-4)

Supplementary methods

Analysis of differential gene expression between *ADAM8/9/15*^{high} and *ADAM8/9/15*^{low} patient samples:

DESeq2: Genes with low counts (rowsums <10 (MMRF); <3 (validation cohort); <1 (siRNA knockdowns); different thresholds due to different sample sizes) were excluded prior to DESeq analysis.

Sample grouping: Patient samples were grouped as *ADAM8/9/15*$^{high/low}$ according to the gene expression of the respective ADAM gene. In the MMRF cohort, the 10% of samples with the highest and 10% of samples with the lowest *ADAM8/9/15* counts (n=92 per condition) were included. In the smaller validation cohort, the highest/lowest 25% (n=18 per condition) were used (see scheme below).

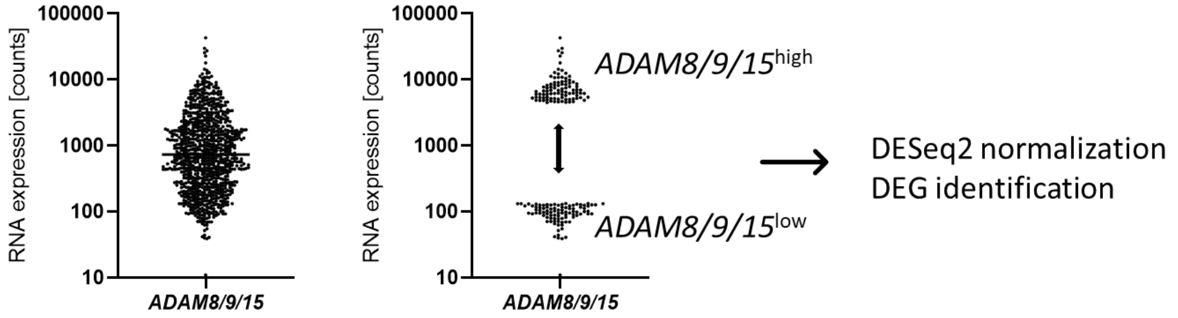

To verify the validity of grouping samples according to their *ADAM8/9/15* GE using counts, we assessed whether the same samples would also be considered as high/low expressors according to normalized counts (*ADAM8/9/15* counts / total amount of counts found in the respective sample) or TPM scores (see Figure below). Samples grouped as *ADAM8/9/15*^{high} according to their counts are marked in red. *ADAM8/9/15*^{low} samples are black. (A-C): MMRF dataset. (D-F): Validation dataset. Groups can be identified as high/low expressors using any of the gene expression measures.

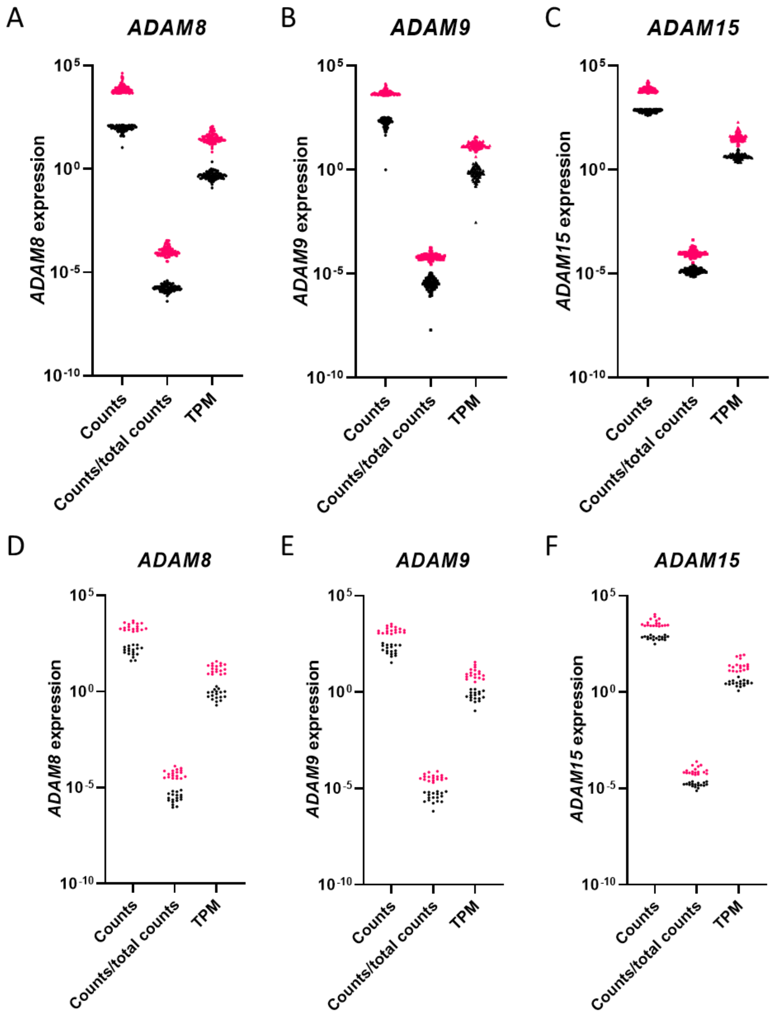

Supplementary tables

Table S1: siRNA sequences and antibodies. siRNAs targeting the maximum amount of transcripts were chosen. BLAST results of targets are summarized in supplementary **Table S2**. All siRNAs are predesigned Silencer Select siRNAs purchased from Ambion by Life technologies (Thermo Fisher Scientific, Waltham, MA, USA).

**siRNAs**			
**Target gene**	**Sense Sequence (5' -> 3')**	**Antisense Sequence 3' -> 5'**	**Catalog #**
ADAM8	GCACCUGCAUGACAACGUAtt	UACGUUGUCAUGCAGGUGCcg	4392420
ADAM9	GCAAACUACUUGGAUAGUAtt	UACUAUCCAAGUAGUUUGCca	4390825
ADAM15	CAUUAUUUCGCGAAUCCAAtt	UUGGAUUCGCGAAAUAAUGgg	4392420
**Primary antibodies**			
**Target protein**	**Dilution**	**Company**	**Catalog #**
ADAM8	1:2 000 in 5 % BSA-TBS-T	Thermo Fisher Scientific, Waltham, MA, USA	MA5 38469
ADAM9	1:4 000 in 5 % milk-TBS-T	Thermo Fisher Scientific	PA5-76732
ADAM15	1:3 000 in 5 % milk-TBS-T	Proteintech, Planegg-Martinsried, Germany	27124-1-AP
IGF1R	1:1 000 in 5 % milk-TBS-T	Cell Signaling Technology (CST), Danvers, MA, USA	9750
pIGF1R (Y1135)	1:1 000 in 5 % BSA-TBS-T	CST	3918
AKT	1:2 000 in 5 % milk-TBS-T	CST	4691
pAKT (S473)	1:1 000 in 5 % BSA-TBS-T	CST	4058
mTOR	1:2 000 in 5 % BSA-TBS-T	CST	2972
pmTOR (S2448)	1:1 000 in 5 % BSA-TBS-T	CST	2971
MYC	1:25 000 in 5 % milk-TBS-T	Abcam, Cambridge, UK	32072
GAPDH	1:100 000 in 5 % BSA-TBS-T	CST	5174
**Secondary antibodies**			
Anti-rabbit	1:2 000 in 5 % milk-TBS-T	CST	7074
Anti-mouse	1:2 000 in 5 % milk-TBS-T	CST	7076

Table S5: Cox proportional hazards model considering high *ADAM8*, *ADAM9* and *ADAM15* GE and the cytogenetic abnormalities associated with a high expression of the respective ADAM gene in the MMRF cohort. PFS: Progression-free survival. OS: Overall survival. HR: Hazard ratio. CI: Confidence interval. (A) Information about *ADAM8* GE, 1q amplification, p53 abnormality and t(4;14) was available for 358 patients. 76 of these patients had an *ADAM8* GE > mean; 1q amplification was detected in 125; p53 abnormality in 64 and t(4;14) in 55 patients. (B) Information about *ADAM9* GE, 1q amplification, t(4;14) and t(14;16) was available for N=494 patients. 206 of these patients had an *ADAM9* GE > mean. The 1q amplification was detected in 188; t(4;14) in 77 and t(14;16) in 46 patients. (C) Information about *ADAM15* GE and 1q amplification status was available for 547 patients. *ADAM15* GE was > mean in 176 of these patients and the 1q amplification was present in 212 patients.

**(A)**	**PFS**			**OS**		
**Variable**	**HR**	**95% CI**	***P*=**	**HR**	**95% CI**	***P*=**
ADAM8 GE>mean	1.43	1.05 - 1.94	**0.0218**	1.74	1.10 - 2.68	**0.0145**
1q_amplification	1.25	0.95 - 1.64	0.1105	1.70	1.13 - 2.54	**0.0103**
p53_abnormality	0.97	0.68 - 1.36	0.8841	0.57	0.29 - 1.01	0.0706
t(4;14)	0.93	0.62 - 1.33	0.6948	0.97	0.53 - 1.66	0.9144
**(B)**	**PFS**			**OS**		
**Variable**	**HR**	**95% CI**	***P*=**	**HR**	**95% CI**	***P*=**
ADAM9 GE>mean	1.44	1.11 - 1.87	**0.0056**	1.91	1.30 - 2.83	**0.0010**
1q_amplification	1.11	0.85 - 1.44	0.4421	1.42	0.96 - 2.10	0.0740
t(4;14)	0.79	0.53 - 1.14	0.2202	0.72	0.40 - 1.24	0.2661
t(14;16)	1.19	0.77 - 1.77	0.4217	1.31	0.68 - 2.32	0.3860
**(C)**	**PFS**			**OS**		
**Variable**	**HR**	**95% CI**	***P*=**	**HR**	**95% CI**	***P*=**
ADAM15 GE>mean	1.58	1.21 - 2.04	**0.0006**	1.98	1.35 - 2.89	**0.0005**
1q_amplification	1.08	0.83 - 1.39	0.5688	1.34	0.91 - 1.96	0.1333

Table S6: Cox proportional hazards model considering high *ADAM8* GE and the cytogenetic abnormality del17p, which was associated with a high expression *ADAM8* in the validation cohort. Information about both *ADAM8* GE and presence of del17p was available for 42 patients. 12 patients had an *ADAM8* GE > mean. Del17p was detected in 12 patients. PFS: Progression-free survival. OS: Overall survival. HR: Hazard ratio. CI: Confidence interval.

	**PFS**			**OS**		
**Variable**	**HR**	**95% CI**	***P*=**	**HR**	**95% CI**	***P*=**
ADAM8 GE > mean	2.55	1.12 to 5.54	**0.0209**	5.18	1.88 to 14.49	**0.0014**
del 17p	1.16	0.52 to 2.45	0.7080	1.03	0.36 to 2.74	0.9590

Supplementary figures

Figure S1: High expression of ADAMs is associated with shorter survival in the validation cohort. (A-C) Correlation of high GE (> mean of all samples) of *ADAM8* (A), *ADAM9* (B) and *ADAM15* (C) with OS in the validation cohort. (D-F) Correlation of high GE (> mean of all samples) of *ADAM8* (D), *ADAM9* (E) and *ADAM15* (F) with PFS in the validation cohort. (G) Since the association between high *ADAM9* GE and PFS was not significant with GE > mean defining GE as high, the analysis was also performed considering a higher threshold (> 10 TPM) as high *ADAM9*. Survival correlations were performed using the Kaplan Meier method and log rank test. n=51 patients. (A, D) *ADAM8* GE > mean: n=15 patients; *ADAM8* GE < mean: n= 36 patients. (B, E) *ADAM9* GE > mean: n=16 patients; *ADAM9* GE < mean: n= 35 patients. *ADAM15* GE > mean: n=13 patients; *ADAM15* GE < mean: n= 38 patients. (G) *ADAM9* GE > 10 TPM: n=5 patients; *ADAM9* GE < 10 TPM: n=46 patients.

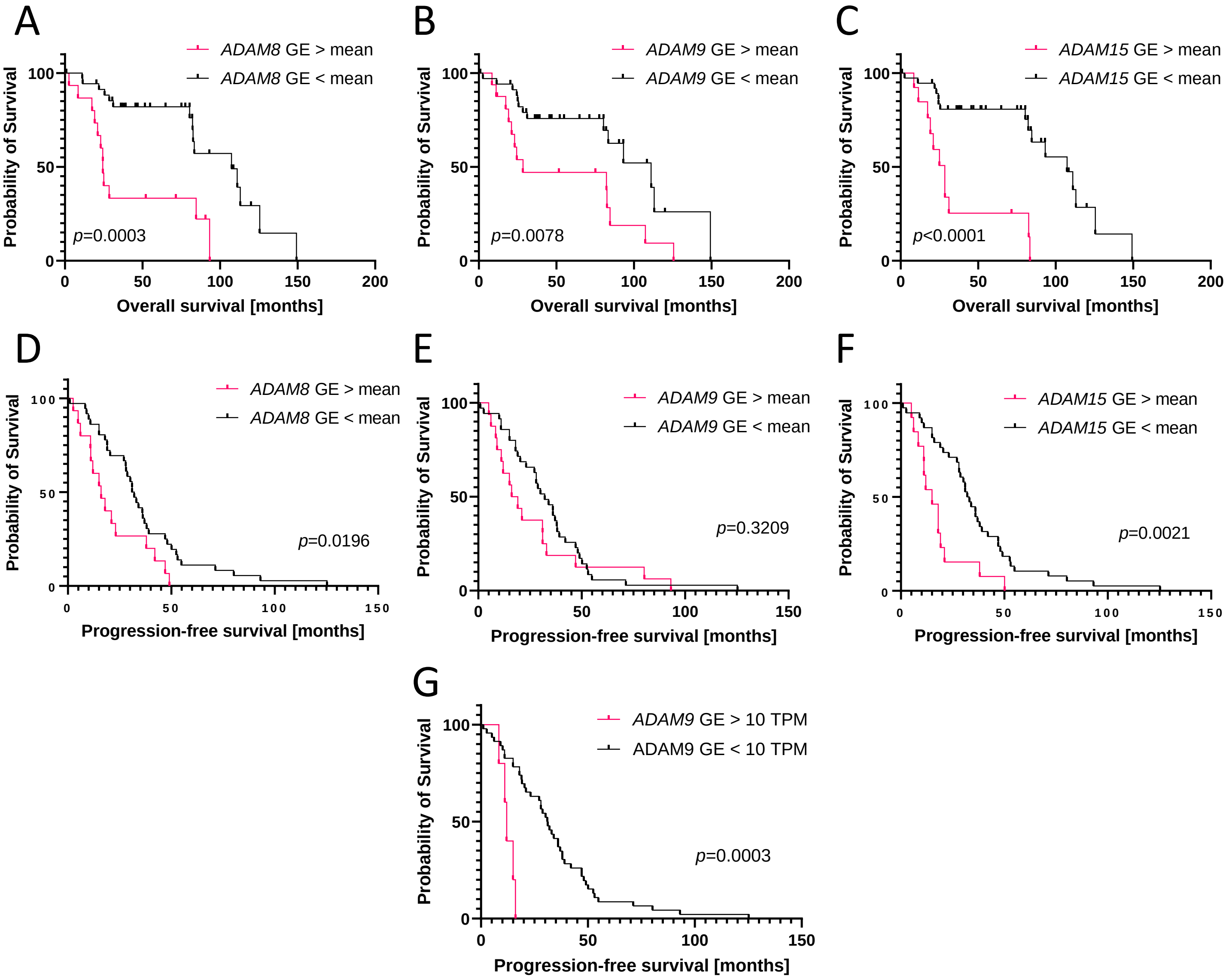

Figure S2: PCA (A,C,E) and volcano plots (B,D,F) for RNA-Seq data concerning *ADAM8* comparisons. PCA was performed on vst transformed data. (A,B): Comparison of *ADAM8*^{high} vs. *ADAM8*^{low} patient samples (top/lowest 10%) from the MMRF cohort. Positive log2fold change signifies upregulation in the *ADAM8*^{high} samples. (C,D): Comparison of *ADAM8*^{high} vs. *ADAM8*^{low} patient samples (top/lowest 25%) from the validation cohort. Positive log2fold change signifies upregulation in the *ADAM8*^{high} samples. (E,F): Comparison of HMCL transfected with scr-siRNA (scr) or siRNA targeting *ADAM8* (si). Negative log2 fold change signifies downregulation in the *ADAM8* siRNA samples.

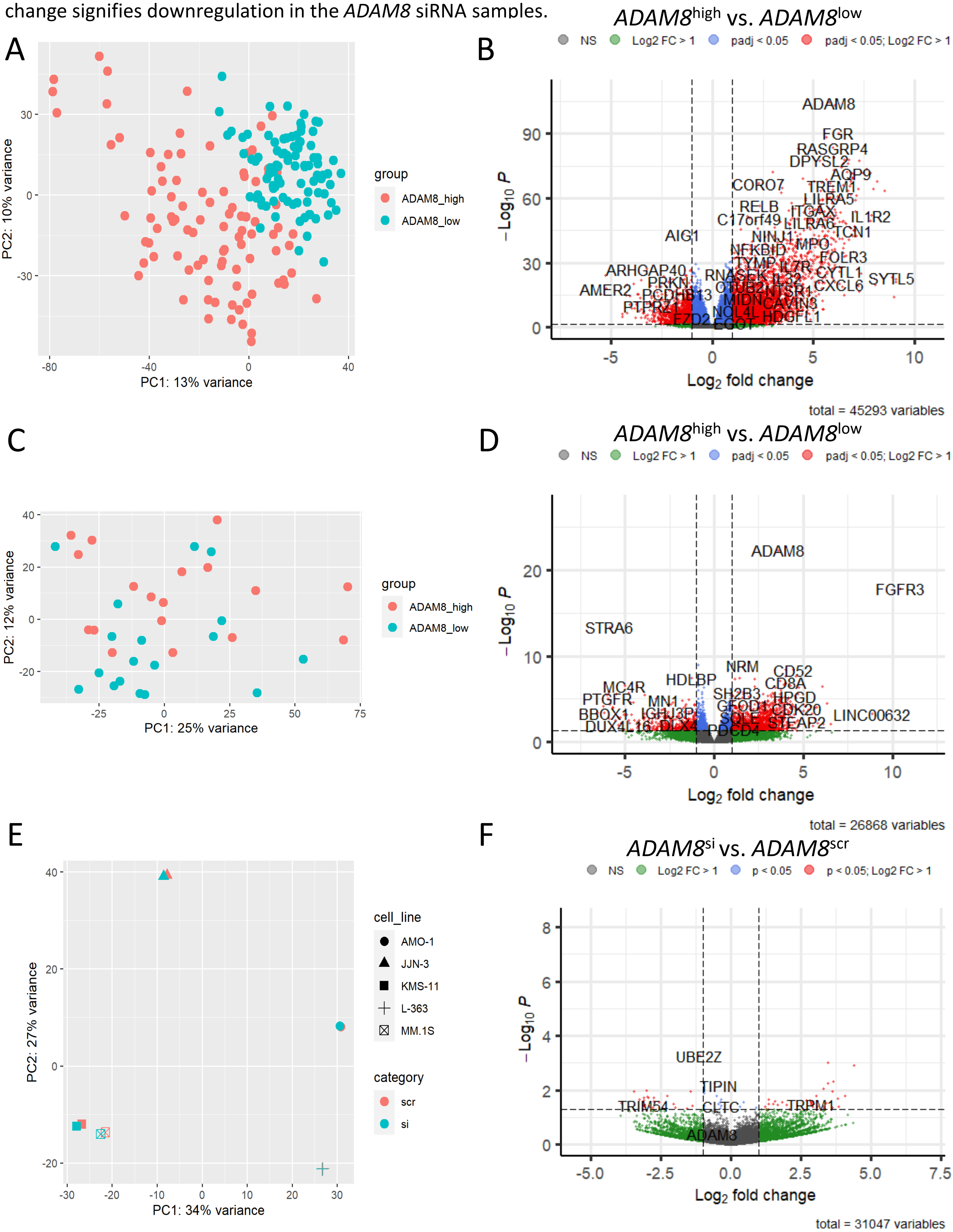

Figure S3: PCA (A,C,E) and volcano plots (B,D,F) for RNA-Seq data concerning *ADAM9* comparisons. PCA was performed on vst transformed data. (A,B): Comparison of *ADAM9*^{high} vs. *ADAM9*^{low} patient samples (top/lowest 10%) from the MMRF cohort. Positive log2fold change signifies upregulation in the *ADAM9*^{high} samples. (C,D): Comparison of *ADAM9*^{high} vs. *ADAM9*^{low} patient samples (top/lowest 25%) from the validation cohort. Positive log2fold change signifies upregulation in the *ADAM9*^{high} samples. (E,F): Comparison of HMCL transfected with scr-siRNA (scr) or siRNA targeting *ADAM9* (si). Negative log2 fold change signifies downregulation in the *ADAM9* siRNA samples.

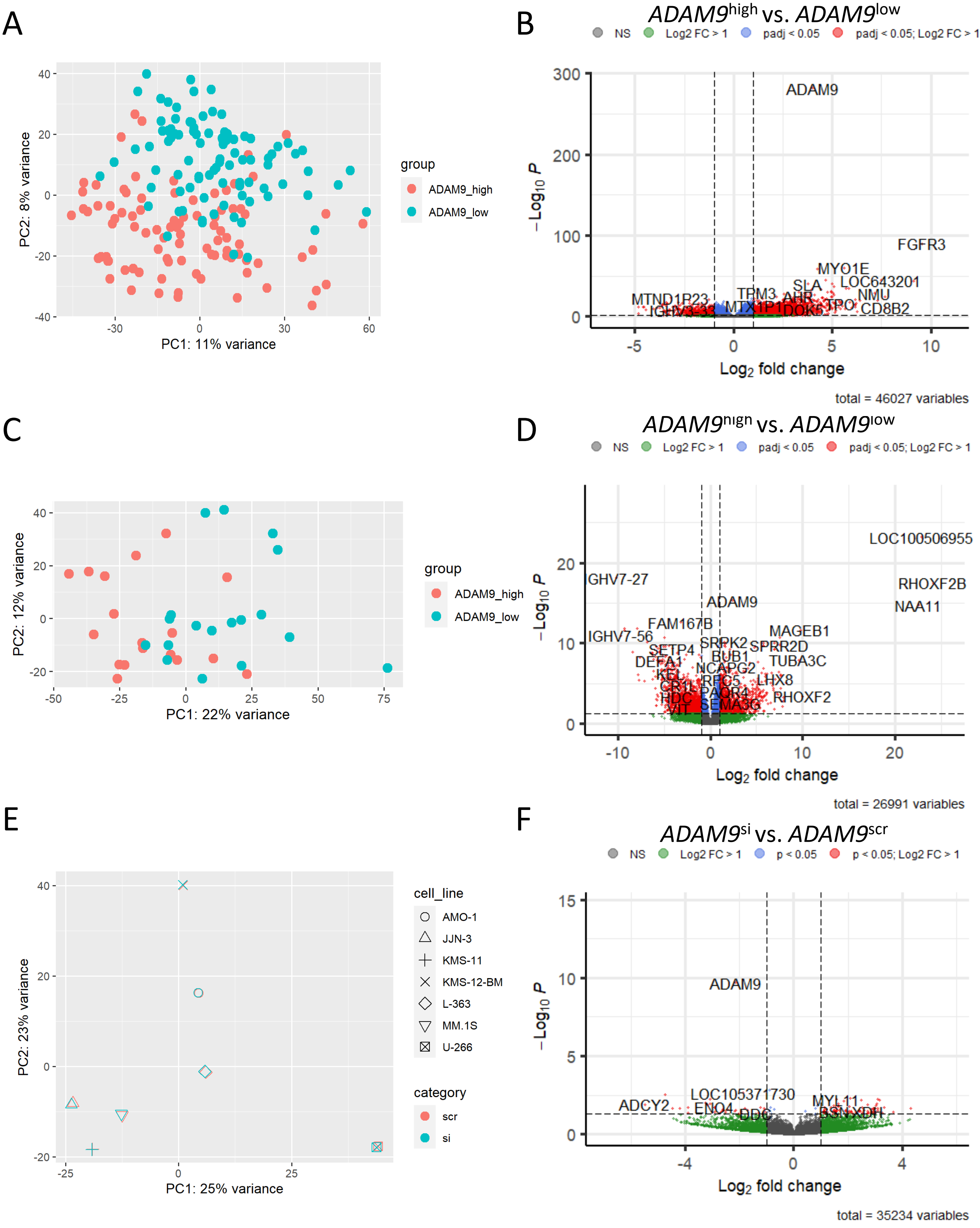

Figure S4: PCA (A,C,E) and volcano plots (B,D,F) for RNA-Seq data concerning *ADAM15* comparisons. PCA was performed on vst transformed data. (A,B): Comparison of *ADAM15*^{high} vs. *ADAM15*^{low} patient samples (top/lowest 10%) from the MMRF cohort. Positive log2fold change signifies upregulation in the *ADAM15*^{high} samples. (C,D): Comparison of *ADAM15*^{high} vs. *ADAM15*^{low} patient samples (top/lowest 25%) from the validation cohort. Positive log2fold change signifies upregulation in the *ADAM15*^{high} samples. (E,F): Comparison of HMCL transfected with scr-siRNA (scr) or siRNA targeting *ADAM15* (si). Negative log2 fold change signifies downregulation in the *ADAM15* siRNA samples.

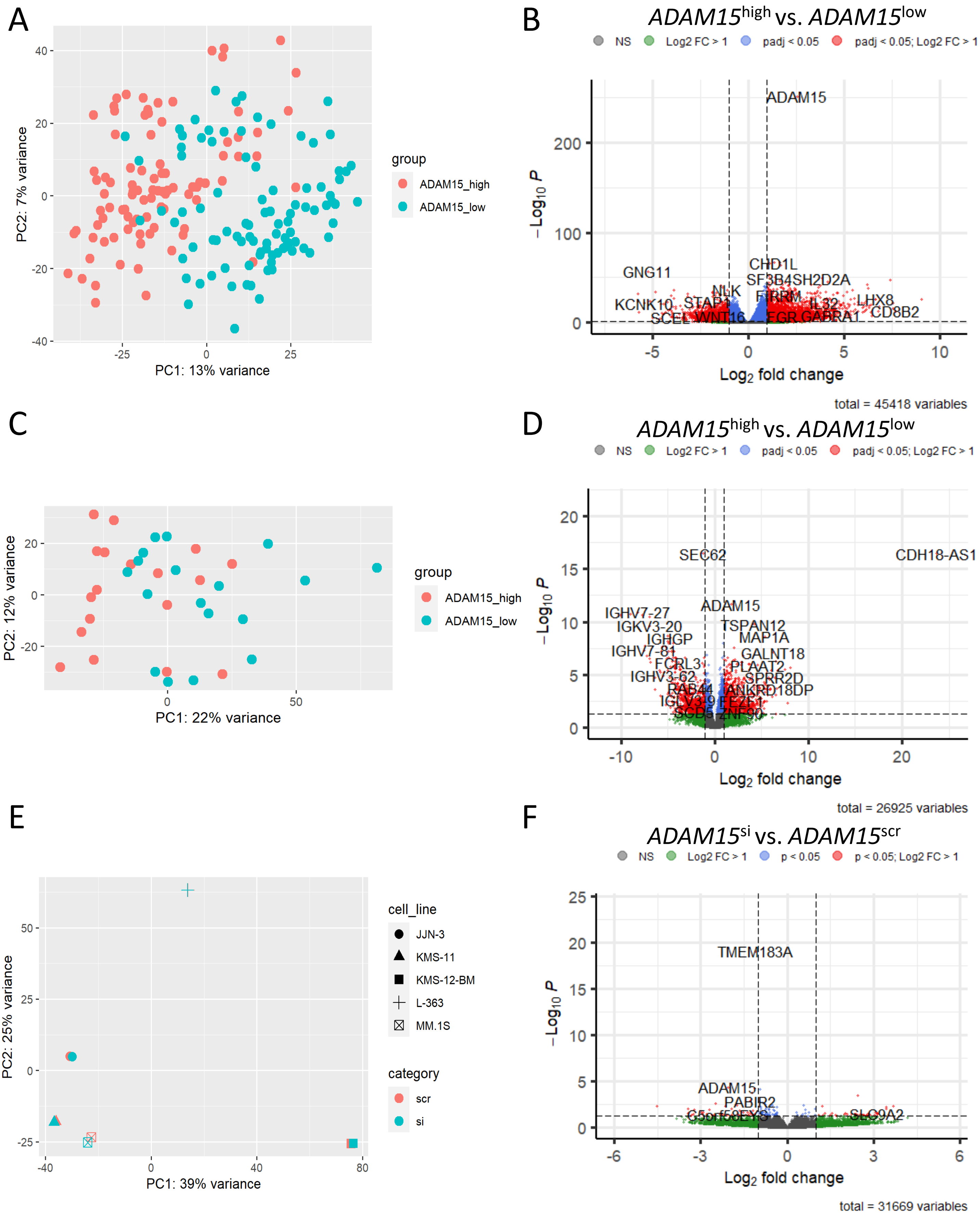

Figure S5: Distribution of samples with and without the translocation t(11;14) in the (A) *ADAM9*$^{low/high}$ and (B) *ADAM15*$^{low/high}$ groups used for DESeq and GSEA in the MMRF and validation cohort (top/lowest 10% or 25% of samples). Analysis includes only cases where both gene expression and translocation status was available. Group sizes are summarized above the bars. Statistical test was Fisher's exact.

A

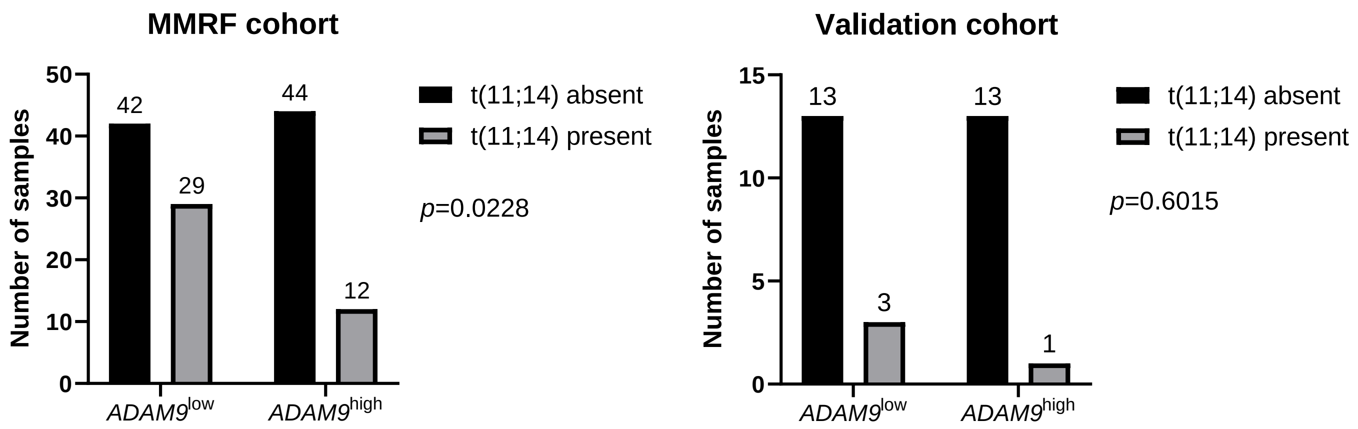

B

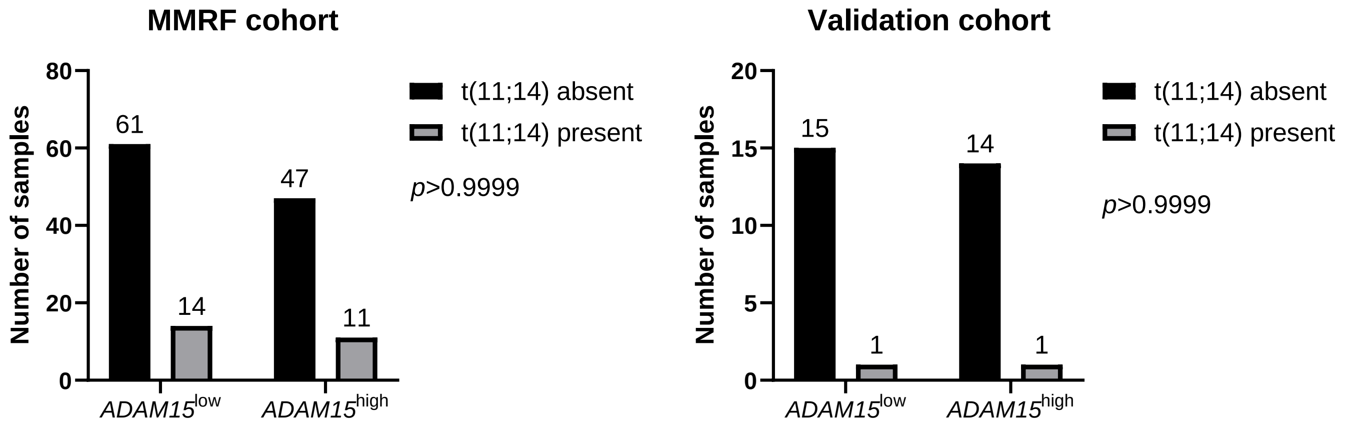

Figure S6: Comparison of (A) Ki67 or (B) MYC protein expression measured by immunohistochemistry (% Ki67- / MYC-positive CD138-positive cells) between *ADAM8/9/15*$^{high/low}$ samples (*ADAM8/9/15 G*E > / ≤ mean of all samples). Statistical test was Mann-Whitney-U test. n=37 for Ki67. n=57 for MYC. Lines show the mean Ki67 or MYC expression in each group.

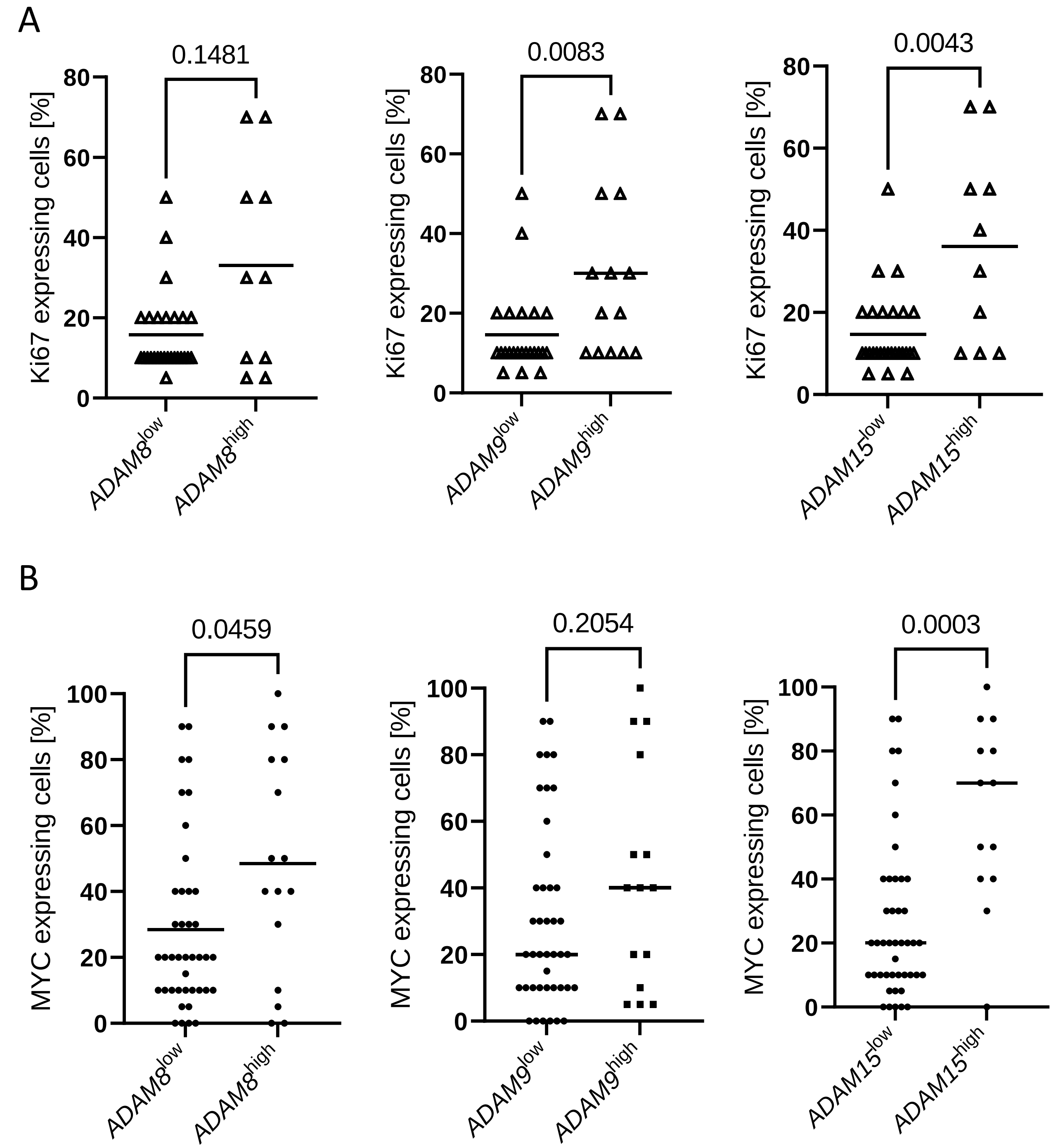

Figure S7: Exemplary immunohistochemical stainings of CD138 and Ki67 in a $Ki67^{high}$ and a $Ki67^{low}$ MM sample from the validation cohort. Samples were classified as $Ki67^{low/high}$ when the sample contained < / ≥ 30% Ki67-positive CD138-positive cells. Pictures were acquired using an OLYMPUS BX50 microscope with 10X magnification.

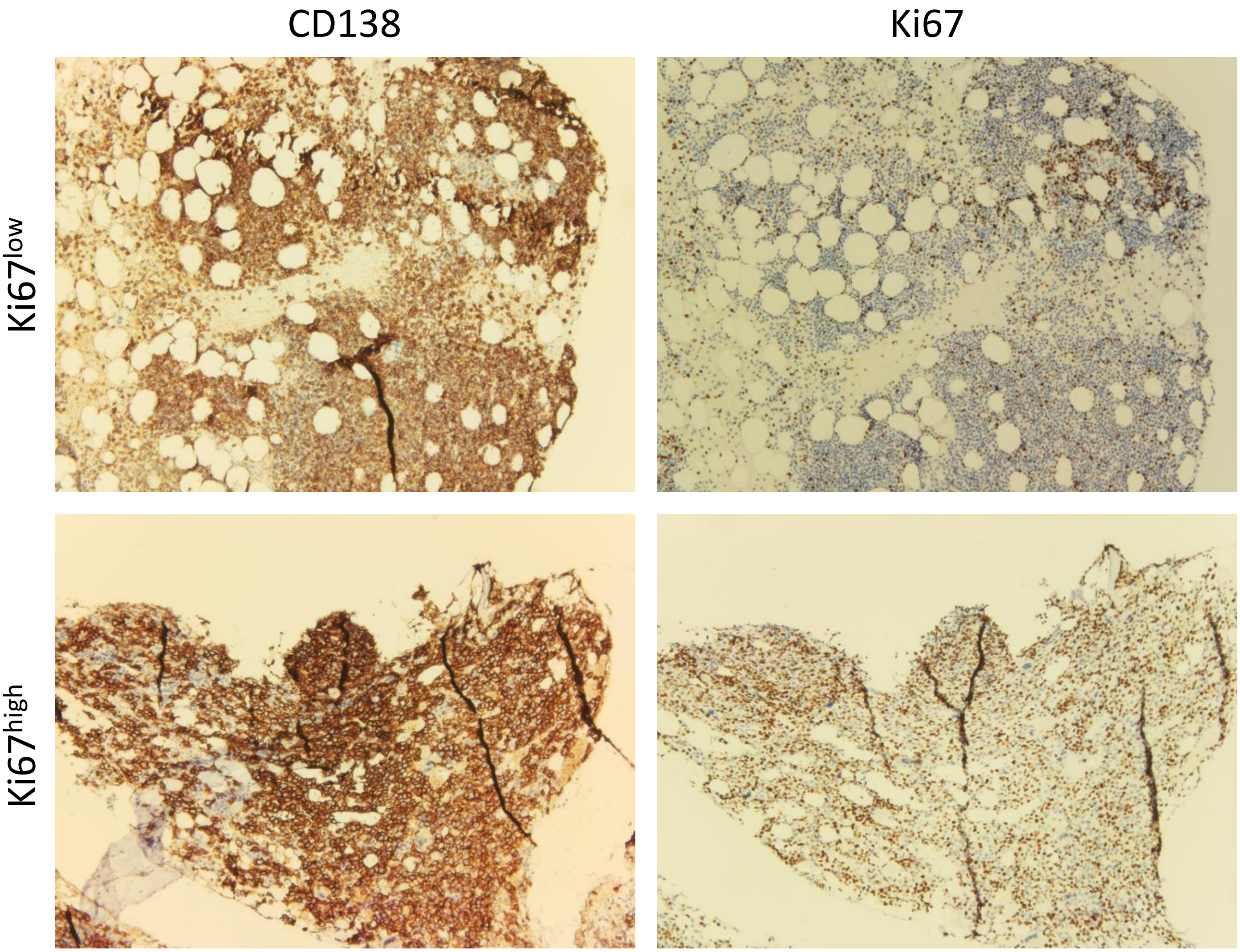

Figure S8: (A, B) Cox proportional hazards model for progression-free (A) and overall survival (B) including *ADAM9* and Ki67 expression status as variables. *ADAM9*^{high}: *ADAM9* GE > mean of all samples. Ki67^{high}: ≥30% Ki67^{+} CD138^{+} cells. (C, D) Since high *ADAM9* GE was only significantly associated with shorter PFS in the univariate analysis at higher thresholds defining *ADAM9* GE as high (> 10 TPM, **Figure S1**), Cox proportional hazards model for progression-free (A) and overall survival (B) was also performed including *ADAM9* and Ki67 expression status as variables with *ADAM9*^{high}: *ADAM9* GE > 10 TPM. Ki67^{high}: ≥30% Ki67^{+} CD138^{+} cells. n=29 patients from the validation cohort. HR: Hazard ratio. CI: Confidence interval.

A

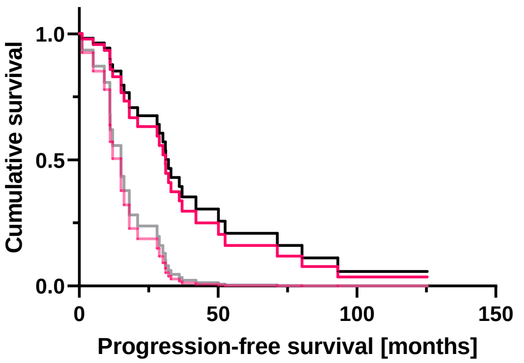

	HR	95% CI	*P* =
ADAM9^{high}	1.17	0.52 – 2.53	0.6968
Ki67^{high}	3.65	1.41 – 8.86	**0.0052**

B

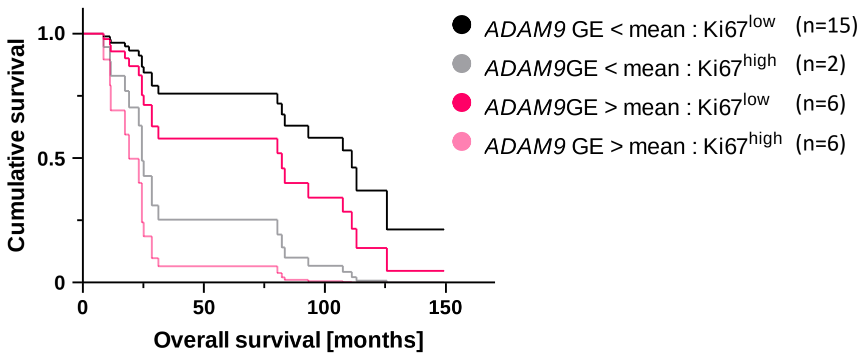

	HR	95% CI	*P* =
ADAM9^{high}	1.99	0.77 – 5.12	0.1495
Ki67^{high}	4.99	1.82 – 13.31	**0.0013**

C

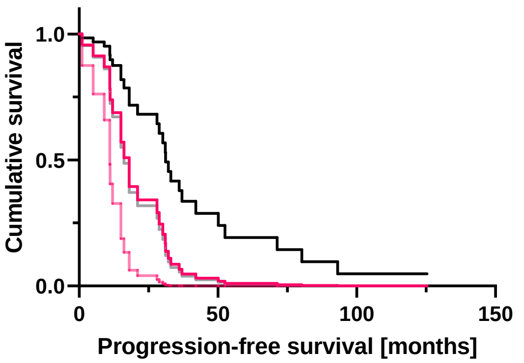

	HR	95% CI	*P* =
ADAM9^{high}	2.80	0.64 – 11.41	0.1530
Ki67^{high}	2.98	1.03 – 7.75	**0.0315**

D

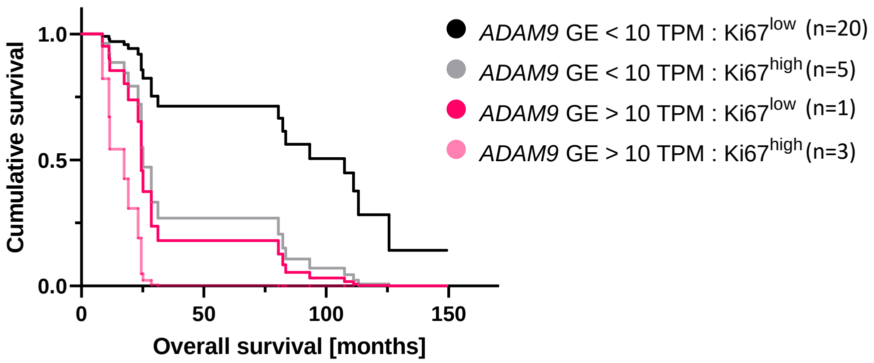

	HR	95% CI	*P* =
ADAM9^{high}	5.08	0.99 – 28.44	0.0508
Ki67^{high}	3.88	1.29 – 10.93	**0.0118**

Figure S9: (A, B) Cox proportional hazards model for progression-free (A) and overall survival (B) including *ADAM8* and Ki67 expression status as variables. n=29 patients from the validation cohort. *ADAM8*^{high}: *ADAM8* GE > mean of all samples. Ki67^{high}: ≥ 30% Ki67^{+} CD138^{+} cells. (C) Cox proportional hazards model for overall survival including *ADAM8* and MYC expression status as variables. n=44 patients. MYC^{high}: ≥ 40% MYC^{+} CD138^{+} cells. Analysis was not performed for PFS because high MYC expression has only been shown to correlate with OS in univariate analyses (see (33)). HR: Hazard ratio. CI: Confidence interval.

A

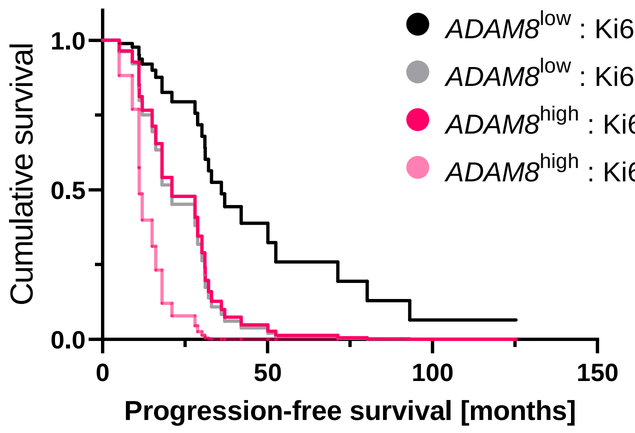

	HR	95% CI	*P* =
ADAM8^{high}	2.56	0.95 – 6.50	0.0533
Ki67^{high}	3.45	1.05 – 7.34	**0.0313**

B

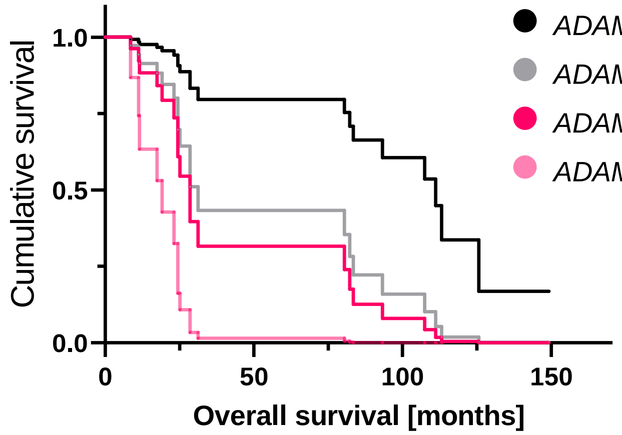

	HR	95% CI	*P* =
ADAM8^{high}	5.05	1.63 - 15.91	**0.0047**
Ki67^{high}	3.67	1.28 - 10.15	**0.0129**

C

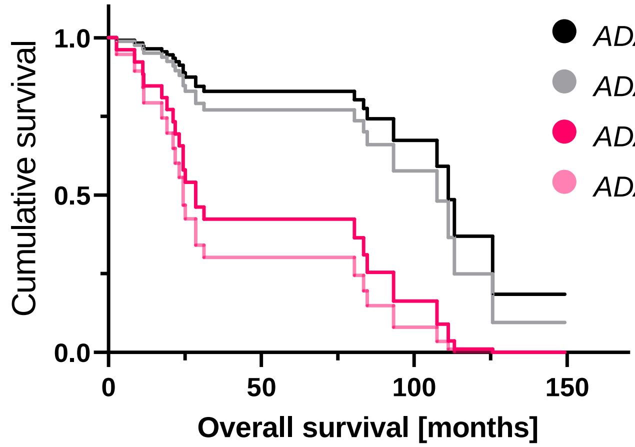

	HR	95% CI	*P* =
ADAM8^{high}	4.59	1.77 - 12.79	**0.0022**
MYC^{high}	1.39	0.58 - 3.50	0.4648

Figure S10: (A, B) Cox proportional hazards model for progression-free (A) and overall survival (B) including *ADAM15* and Ki67 expression status as variables. n=29 patients from the validation cohort. *ADAM15*^{high}: *ADAM15* GE > mean of all samples. Ki67^{high}: ≥ 30% Ki67^{+} CD138^{+} cells. (C) Cox proportional hazards model for overall survival including *ADAM15* and MYC expression status as variables. Analysis was not performed for PFS because high MYC expression has only been shown to correlate with OS in univariate analyses (see (33)). n=44 patients. MYC^{high}: ≥ 40% MYC^{+} CD138^{+} cells. HR: Hazard ratio. CI: Confidence interval.

A

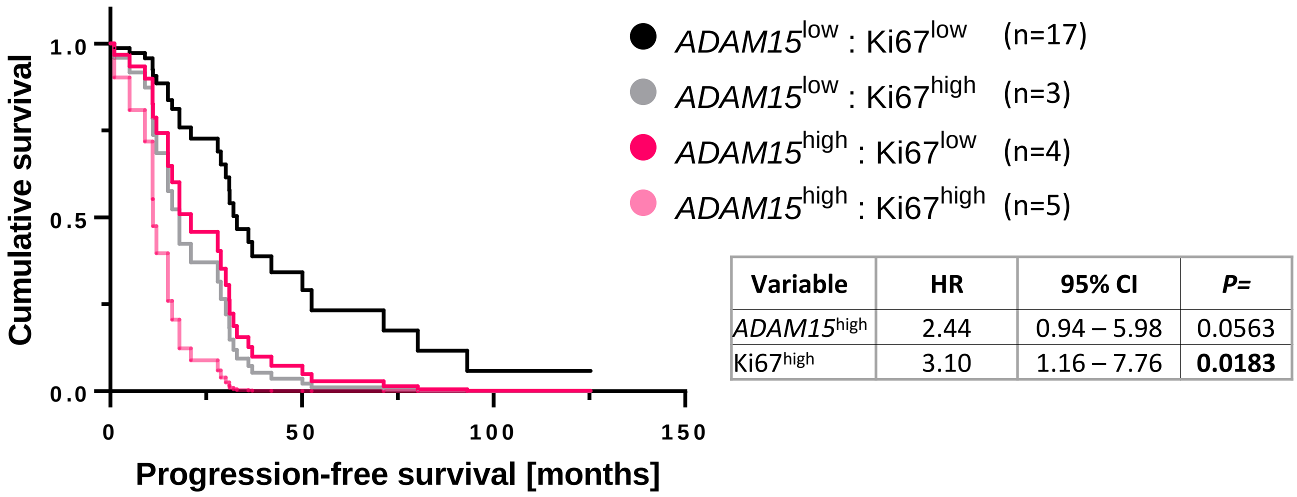

Variable	HR	95% CI	*P=*
ADAM15^{high}	2.44	0.94 – 5.98	0.0563
Ki67^{high}	3.10	1.16 – 7.76	**0.0183**

B

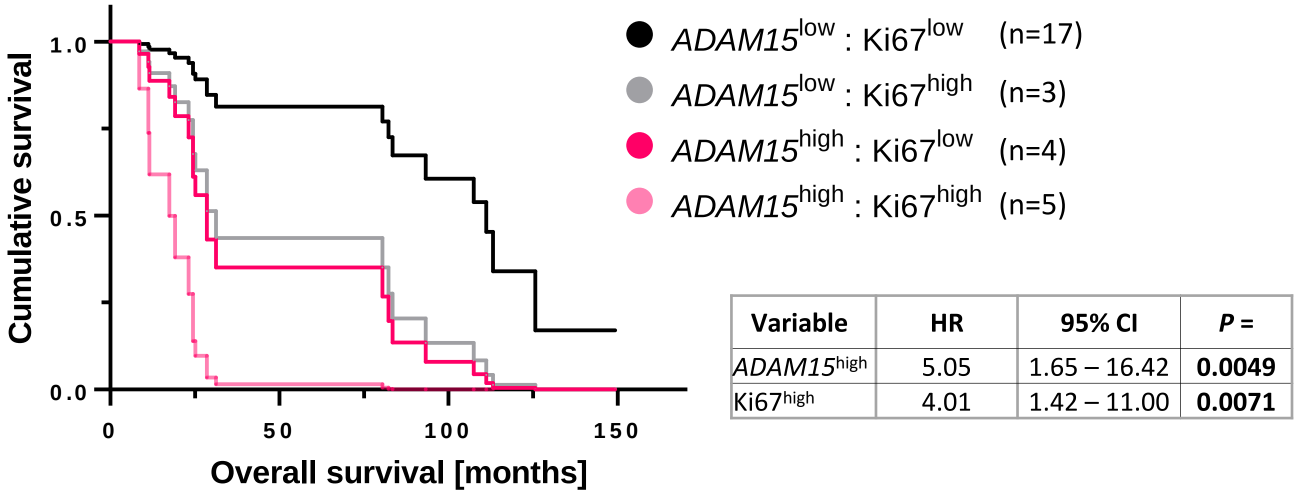

Variable	HR	95% CI	*P =*
ADAM15^{high}	5.05	1.65 – 16.42	**0.0049**
Ki67^{high}	4.01	1.42 – 11.00	**0.0071**

C

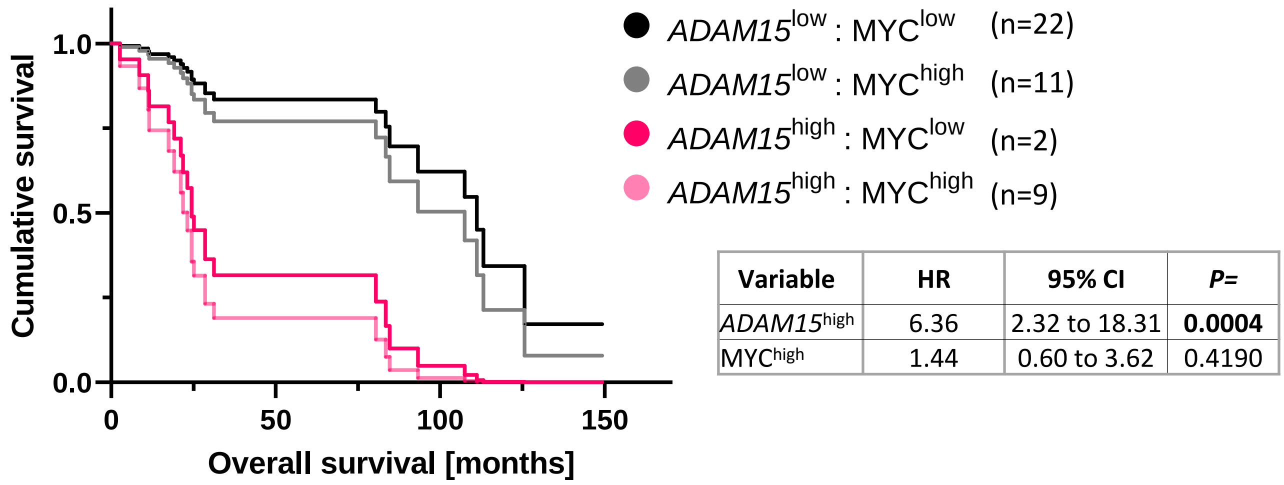

Variable	HR	95% CI	*P=*
ADAM15^{high}	6.36	2.32 to 18.31	**0.0004**
MYC^{high}	1.44	0.60 to 3.62	0.4190

Figure S11: ADAM8, ADAM9 and ADAM15 expression in HMCL. 20 µg protein were used and representative WB of three independent rounds of experiments is shown. Antibody specificity was verified by siRNA knockdowns in HMCL. Bands that were downregulated by the siRNA knockdown are marked with a red <. Ab: antibody.

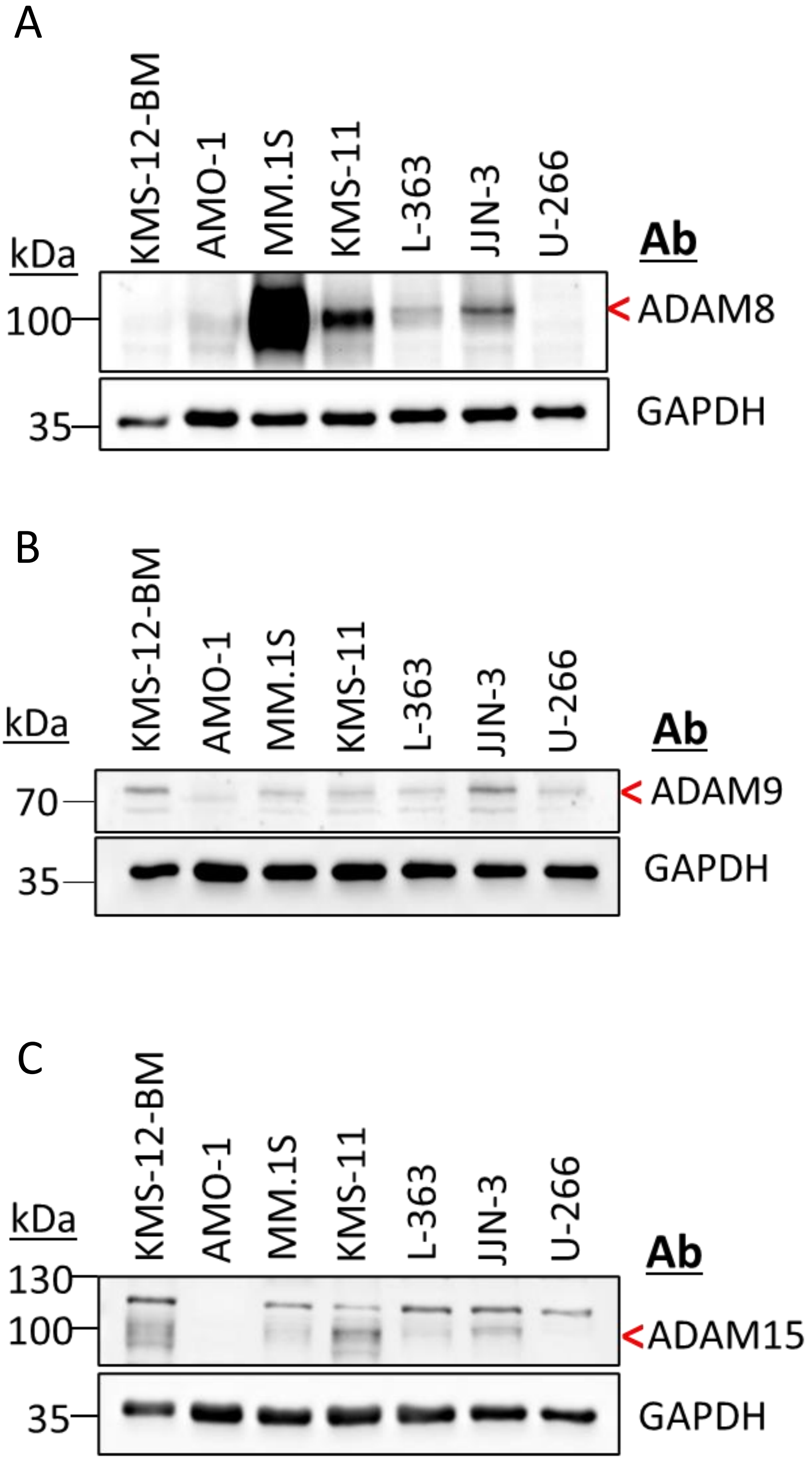

Figure S12: Summary of significantly enriched gene sets for the comparison of HMCL before (scr-siRNA transfected) and after *ADAM8/9/15* siRNA knockdown. Petrol bars show gene sets that were also enriched in the comparison of *ADAM8/9/15*[high] and *ADAM8/9/15*[low] patient samples from the MMRF and/or validation cohort (**Table S8**).

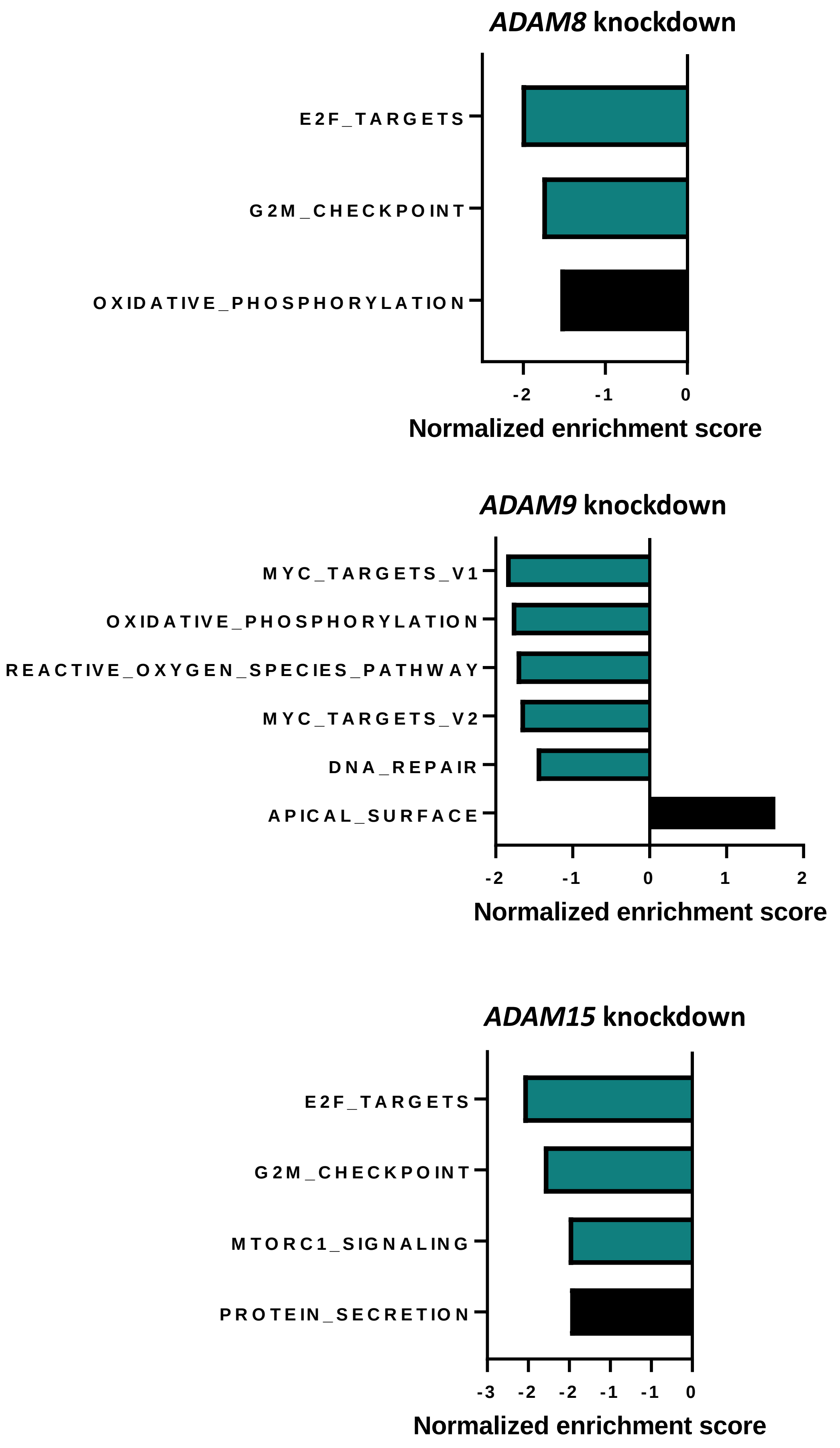

Supplement: Supplementary file 1 — Supplementary methods, figures and tables S1, S5, S6 [file 41408_2024_1133_MOESM1_ESM.pdf]
